# Ultrahigh-speed chromatically band-limited illumination profilometry

**DOI:** 10.1038/s41467-026-78478-w

**Published:** 2026-10-12

**Authors:** Cheng Jiang, Jinyang Liang

**Affiliations:** Laboratory of Applied Computational Imaging, Centre Énergie Matériaux Télécommunications, Institut National de la Recherche Scientifique, Université du Québec, Varennes, QC Canada

**Keywords:** Imaging and sensing, Optical metrology, Imaging techniques, Applied optics

## Abstract

Single-shot fringe projection profilometry (SS-FPP) has been extensively applied to the study of rapid three-dimensional (3D) dynamics in physics, engineering, and biology. Despite these advancements, the imaging capability of time-resolved SS-FPP is still limited by the flexibility and speed of both pattern projection and image acquisition, as well as by the duration of the observation time window. Here, we overcome these constraints by developing chromatically band-limited illumination profilometry (C-BLIP). On the illumination side, C-BLIP projects programmable grayscale fringes at ultrahigh speed by synergizing a digital micromirror device with a user-selectable encoding region of interest and a custom-designed projection lens for chromatically band-limited illumination with incoherent light sources. On the detection side, C-BLIP acquires spatially multiplexed color fringe images, followed by 3D image reconstruction. Using C-BLIP, we demonstrated color 3D imaging of a turbofan blade-projectile collision at 25 thousand frames per second (kfps), as well as intensity 3D imaging of maple samara’s fall aerodynamics at 75 kfps and ultrasound encryption/recovery at 150 kfps. These results highlight C-BLIP’s promise for continuous-streaming, ultrahigh-speed 3D metrology in industrial inspection, bionics, and data security.

## Abstract (web summary)

Seeing 3D dynamics requires ultrahigh-speed profilometry. Here, authors develop chromatically band-limited illumination profilometry for continuous-streaming 3D surface imaging with a record speed of 150 thousand frames per second for applications in industrial inspection, bionics and data security.

## Introduction

Ultrahigh-speed three-dimensional (3D) imaging in real time (i.e., during the occurrence of the event) is essential for comprehensively understanding non-repeatable transient phenomena across numerous fields, including biomedicine, autonomous driving, and surface metrology^1–3^. Among existing methods, fringe projection profilometry (FPP) is widely used for its non-contact and high-accuracy measurement capabilities^4,5^. FPP typically works by illuminating a 3D object with spatially periodic fringes and then analyzing the deformed structure image to recover the 3D surface information^6,7^. FPP is mainly propelled by innovations in fringe generation (Supplementary Note 1) and phase extraction (Supplementary Note 2). Early FPP used physical gratings (e.g., moiré profilometry^8^), whereas modern systems commonly rely on digitally generated fringe patterns displayed by programmable light projectors (e.g., computer-generated moiré profilometry^9^ and video projection-based profilometry^10^). Phase information is extracted from either multiple or a single captured image. Multi-frame methods (e.g., phase-shifting profilometry^11^, modulation measuring profilometry^12^, and phase-differencing profilometry^13^), accompanied by temporal phase unwrapping^14,15^, have been implemented in large-depth-range and high-reflectivity-difference imaging conditions^16,17^. Single-frame methods [e.g., empirical mode decomposition-based profilometry^18^ and Fourier transform profilometry (FTP)^19^], teaming up with spatial phase unwrapping^20–23^, are highly robust to motion blur. Further enhanced by deep learning^24–26^, single-shot FPP (SS-FPP) has been established as one of the leading techniques in ultrahigh-speed 3D imaging^27,28^.

Despite these salient merits, time-resolved SS-FPP leaves ample room for improvement in optical instrumentation. Early attempts relied on high-speed projection with ordinary cameras. In such systems, a dominantly used component is the digital micromirror device (DMD), which provides programmable binary pattern projection at tens of kHz^29,30^. By controlling the system’s bandwidth via spatial filtering^31–34^ or defocusing^35–37^, one binary DMD mask can be converted into one grayscale sinusoidal pattern, facilitating accurate phase extraction for 3D profilometry. Leveraging DMD’s flexibility, several light tags—including spatial frequency^38,39^, fringe orientation^40,41^, color^42,43^, and polarization^44,45^—have been exploited to record multiple fringe images into a single camera snapshot. By properly matching the projection rate to the camera’s acquisition rate, 3D images can be continuously streamed. Nonetheless, the imaging speed remains capped at ~22 thousand frames per second (kfps)^46^, insufficient to capture many transient dynamics with microsecond-scale temporal features. Moreover, filtering-based spatial bandwidth control requires coherent light sources, which is less user-friendly for many industrial or biomedical settings^47,48^. Finally, defocusing-based spatial bandwidth control inevitably introduces phase distortion^49^ and pattern-quality instability over fringes with different spatial frequencies^50^, both of which may degrade measurement accuracy.

To overcome these limitations, recent advances have focused on accelerating 3D imaging via high-speed detection. A notable example is compressive phase-shifting (CPS) FPP^51,52^, which integrates multiple spatially encoded fringe images into a single snapshot. A sequence of 3D images is reconstructed by a compressed-sensing algorithm. While CPS-FPP holds promise for time-resolved imaging, its spatial encoding operation can reduce spatial resolution. This modality also depends on intensive computation and scene sparsity for accurate reconstruction, potentially limiting its robustness and reliability in dynamic or highly complex environments. To relax the requirements in computational imaging, single-shot super-resolved FPP^53^ employed two ultrahigh-speed monochromatic cameras for 3D imaging at up to 100 kfps. However, most of these cameras rely on internal memory to store captured frames, preventing continuous-streaming 3D imaging. Moreover, such cameras are often prohibitively expensive for typical research and industrial laboratories, severely limiting their widespread adoption in practical applications.

To address these challenges, we develop chromatically band-limited illumination profilometry (C-BLIP) for multiscale ultrahigh-speed 3D surface imaging. We design and fabricate a projection lens with chromatically varied spatial bandwidth to generate grayscale sinusoidal fringes from a binary pattern illuminated by incoherent light, eliminating the need for spatial filtering or image defocusing. We also display spatial sinusoids at different orientations in a user-selectable encoding region of interest (ROI) on a DMD, thereby allowing for image multiplexing with a cost-effective machine-vision camera. The resulting C-BLIP system enables continuous streaming of color 3D images at 25 kfps and intensity 3D images at up to 150 kfps.

## Results

### Operating principle

**Fig. 1:**
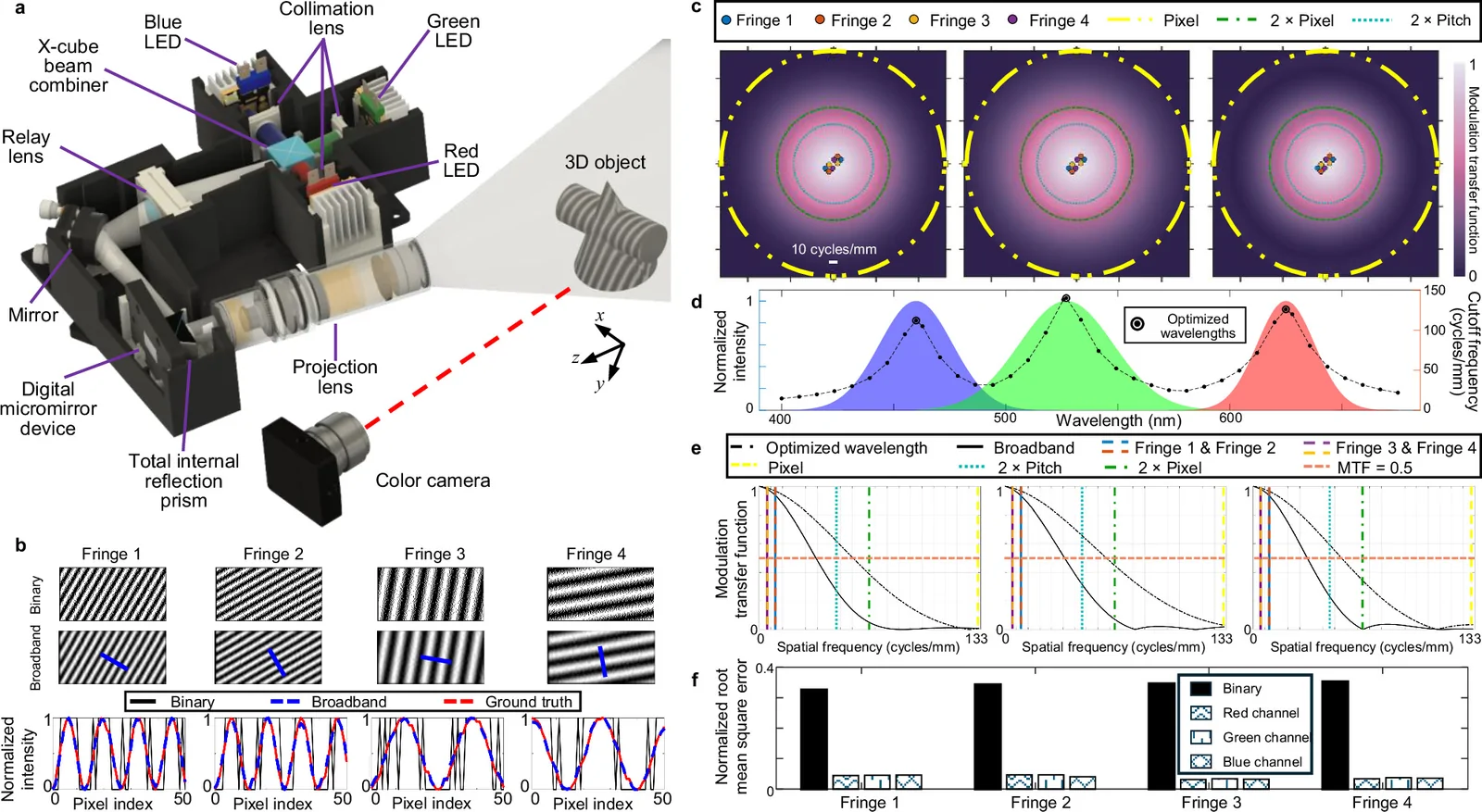
Operating principle of chromatically band-limited illumination profilometry (C-BLIP). **a** Optical layout of the C-BLIP system. The CAD models are courtesy of Luminus Devices, Inc. (for the LEDs), Thorlabs, Inc. (for the mirror mount, lenses, lens tubes, optic spacers, and mount adapters), and Edmund Optics, Inc. (for the lenses). **b** Illustration of a 128×128-pixel local region of error‑diffused binary DMD masks (top) and generated grayscale sinusoids with broadband illumination of the green light-emitting diode (LED) (middle). The bottom row shows the comparison of line profiles marked by the blue lines in the middle row. **c** Modulation transfer functions (MTFs) optimized for the central wavelengths of red (left), green (middle), and blue (right) LEDs. **d** LED emission spectra (shaded) overlaid with the wavelength-dependent cutoff frequencies. **e** Comparison of optimized monochromatic MTFs (dash-dotted lines) and the corresponding broadband counterparts (solid lines). **f** Normalized root mean square error of the error‑diffused binary DMD masks and generated grayscale patterns with respect to their corresponding sinusoids.

A schematic of the C-BLIP system is shown in Fig. 1a with an animated illustration in Supplementary Movie 1. Three light-emitting diodes (LEDs, CBT-90TE, Luminus)—with central wavelengths of 460 nm, 527 nm, and 620 nm and spectral bandwidths of 50–90 nm—serve as the light source. Emitted light is collimated individually, then combined using an X‑cube beam combiner (P-RGB-C, WTS Photonics), and relayed onto a DMD (AJD4500, Ajile Light Industries) via a 50-mm lens (AC254-050-A, Thorlabs) and a total internal reflection prism (TIR690, Sicube Photonics). As shown in Fig. 1b, the DMD displays a sequence of four error‑diffused binary fringe patterns with a period of 97.2 µm oriented at 30° and 60° (denoted as Fringe 1 and Fringe 2) and with a period of 194.4 µm oriented at 10° and 80° (denoted as Fringe 3 and Fringe 4). These binary patterns pass through a chromatically band-limited projection lens designed and fabricated in-house. This customized projection lens leverages dispersion from the selected singlet lens elements to the broad spectral bands of the employed three LEDs while optimizing the MTF only for the central wavelengths of these LEDs (Fig. 1c–e, detailed in Methods, Supplementary Note 3, Supplementary Figs. 1–2, and Supplementary Table 1). The restricted spatial bandwidth to non-central wavelengths in LED illumination acts as smooth-edged low-pass filters that convert binary DMD masks into high-quality grayscale fringe patterns projected onto the 3D objects (Fig. 1b, f). These patterns are distorted by the object’s 3D geometry, and the resulting deformed fringe images are captured by a camera lens (AZURE-3520MX5M, AZURE Photonics) connected to a CoaXPress color camera (Cyclone-2-2000-C, Optronis GmbH). Each captured frame is separated into its RGB channels and demultiplexed using spatial frequency filtering. The output fringe images are then individually processed using the FTP method to extract the phase information (detailed in Methods and Supplementary Fig. 3). Finally, the 3D information is reconstructed by using the triangulation method with the system calibration parameters (detailed in Methods and Supplementary Note 4).

C-BLIP supports two operating modes. In the color-3D-imaging mode, all three LEDs are illuminated simultaneously, providing continuous-wave white-light illumination. In the intensity-3D-imaging mode, the three LEDs illuminate the object sequentially within the display time of each DMD pattern. In both modes, the DMD’s refresh rate is determined by the user-selectable pattern size, and the color camera is operated at one-quarter of the DMD’s refresh rate. System synchronization and specifications are detailed in Supplementary Note 5 and Supplementary Table 2. Depth resolution quantification is provided in Supplementary Note 6 and Supplementary Fig. 4.

C-BLIP overcomes several key bottlenecks of existing time-resolved SS-FPP. Its image acquisition framework, paired with a continuous-streaming machine-vision camera, removes the limited time window. Its image recovery paradigm, using a computationally lightweight phase retrieval technique, avoids iterative image reconstruction. The flexible combination of tri-color LED illumination along with the projection patterns and user-selectable encoding ROI on the DMD equips C-BLIP to address diverse imaging tasks. In the following subsections, we will first validate C-BLIP’s color 3D imaging fidelity using static optical illusions. Then, we will demonstrate C-BLIP’s utility by capturing a turbofan blade-projectile collision at 25 kfps, mapping maple samara’s fall aerodynamics at 75 kfps, resolving encrypted ultrasound signals at 150 kfps, and recovering audio from ultrasound-induced vibration at 150 kfps.

### Unveiling optical illusions

**Fig. 2:**
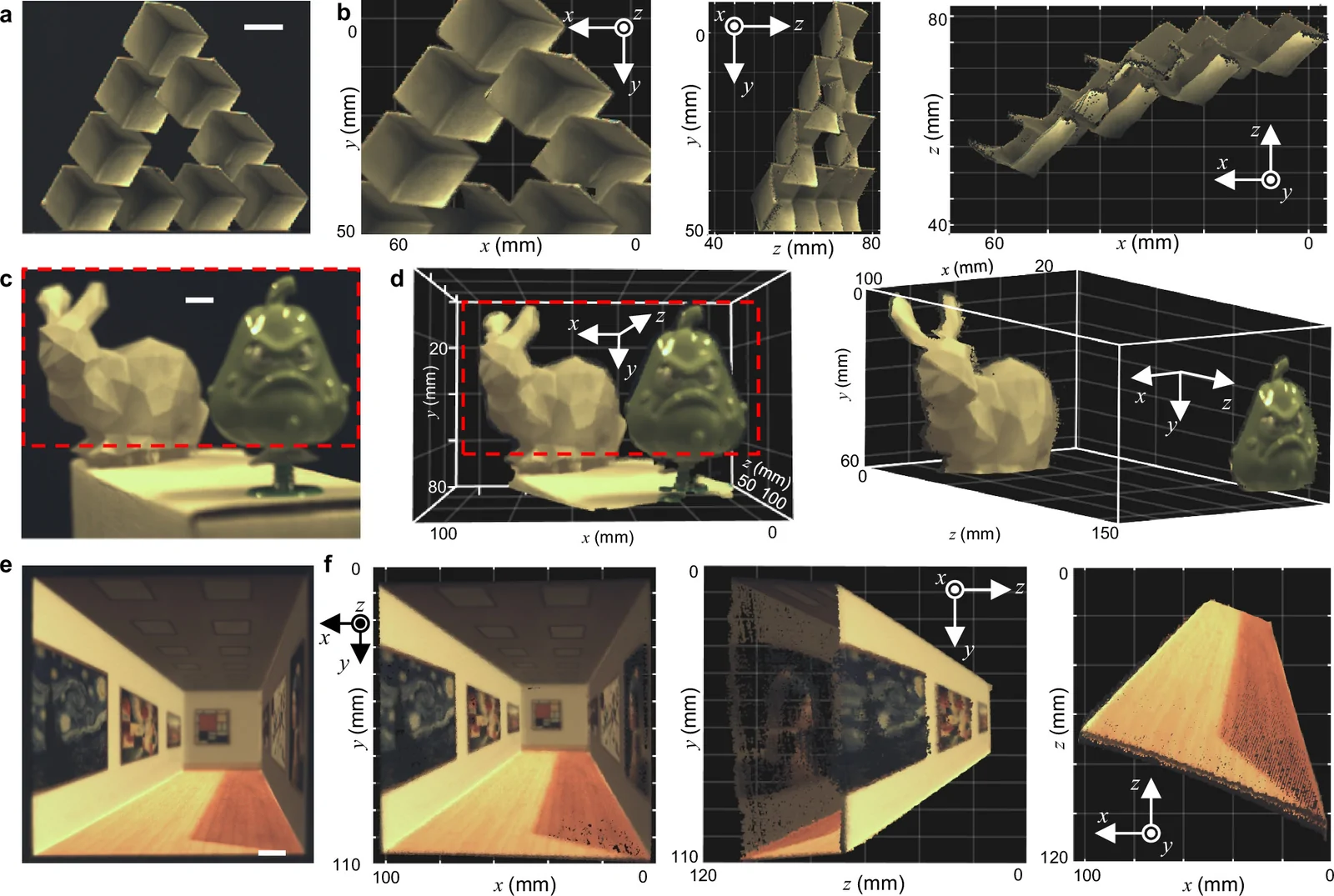
Unveiling optical illusions using C-BLIP. **a**,**b** 2D photograph (**a**) and 3D reconstruction by C-BLIP (**b**) of a half-cube-array illusion. **c**,**d** 2D photograph (**c**) and 3D reconstruction by C-BLIP (**d**) of a size-distance illusion. The region of interest is marked by the red dashed box. **e**,**f** 2D photograph (**e**) and 3D reconstruction by C-BLIP (f) of a depth illusion. Scale bars in (**a**), (**c**), and (**e**): 10 mm.

To validate C-BLIP’s accurate color 3D imaging ability, we used it to reveal optical illusions related to morphology, size, and depth (Supplementary Movie 2). The first demonstration involved a white half‑cube array. When viewed head‑on, each element appears as a complete cube, and the three sides of the triangular structure seem to change continuously in depth (Fig. 2a). In contrast, the 3D image from C-BLIP (Fig. 2b) reveals that the front faces are absent. Next, C-BLIP was deployed to image a 4.5 cm‑tall toy pear and a 6.5 cm‑tall rabbit statue positioned at different distances to appear approximately the same size in the 2D image (Fig. 2c). The reconstruction shown in Fig. 2d clearly recovers the true sizes of both objects and their actual depth relationship. Finally, we tested a pyramid‑based perspective illusion that appeared to depict a corridor receding into the background (Fig. 2e). The C-BLIP imaging results inverted this perception, revealing a protruding center (Fig. 2f). These experiments confirm C-BLIP’s fidelity in recovering complex color 3D geometry.

### Color 3D imaging of a turbofan blade-projectile collision at 25 kfps

**Fig. 3:**
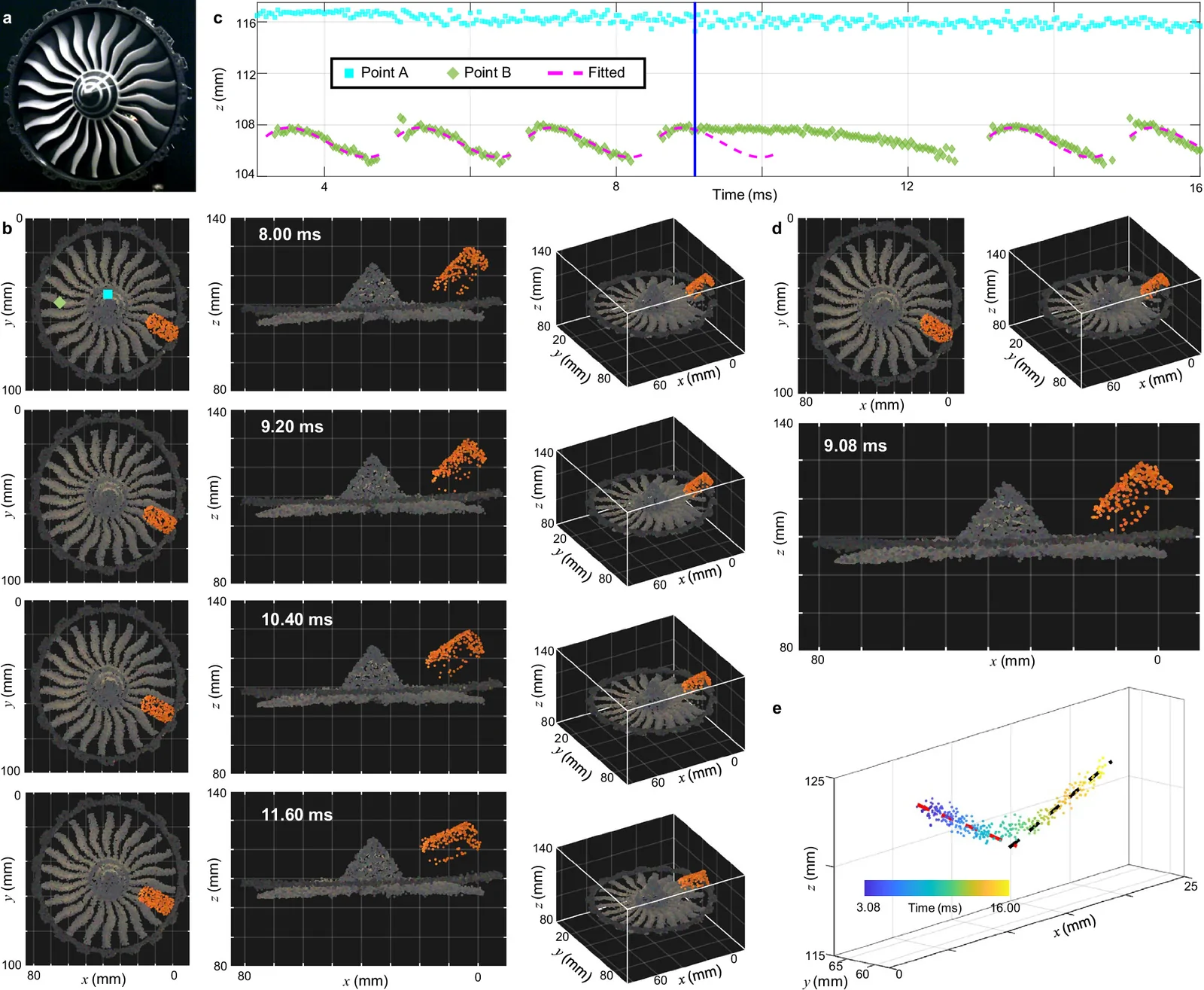
C-BLIP of a turbofan blade-projectile collision. **a** 2D photograph of a turbofan toy. **b** Representative 3D reconstructions of the rotating blades colliding with an earplug projectile at four time points, showing from the front view (left column), the side view (middle column), and an oblique view (right column). **c** Time history of two selected points in (**b**), marked by the aqua square and the green diamond. Magenta dashed line: third-order polynomial fit to the blade-sweep depth. Blue solid line: occurrence of collision. **d** Reconstructions at the collision instant (t = 9.08 ms), showing in the same three viewing angles as in (**b**). **e** Trajectory of the earplug’s centroid. Red and black dashed lines: linear fits to the pre-impact and post-impact paths, respectively.

To demonstrate C-BLIP’s ultrahigh‑speed color 3D imaging, we imaged the collision of an orange earplug used as a projectile against a white toy turbofan rotating at ~1600 revolutions per minute (RPM) (Fig. 3a). The DMD was operated at 25 kHz by using an encoding ROI of 160 × 1140 pixels, with a depth resolution of 0.70 ± 0.08 mm (mean ± standard deviation). The full evolution is provided in Supplementary Movie 3. As shown in the reconstructions from three viewing angles (Fig. 3b), the earplug approaches the turbofan in a head-on orientation, with its tip facing the blade. Upon impact, it rebounds and flips, with its tip facing away from the blade. Displayed in Fig. 3c, the time-resolved depth traces of two selected points reveal the stationary toy turbofan housing and successive blade sweeps with a period of 1.73 ms. Given that the turbofan has 22 blades, the full rotation period was calculated to be 38.1 ms, yielding a rotational speed of 1578 RPM. A third-order polynomial fit (magenta dashed line) accurately describes the first three blade sweeps starting at t = 3.32 ms, but diverges sharply at t = 9.08 ms, marking the collision instant (Fig. 3d). The disturbance lasts until t = 10.80 ms, after which the fan resumes an almost regular rotation. By tracking the earplug’s geometric centroid (Fig. 3e), we resolved the approach, collision, and post‑impact motion. The analysis shows that the earplug had a pre-impact (i.e., t < 9.08 ms) speed of 0.54 m/s. During the impact interval (i.e., t = 9.08–10.80 ms), the centroid depth remains nearly constant, indicating that the earplug is momentarily compressed while interacting with the blades. The post-impact speed increased to 1.03 m/s, indicating strong energy transfer from the blade to the earplug.

### Intensity 3D imaging of falling maple samaras at 75 kfps

**Fig. 4:**
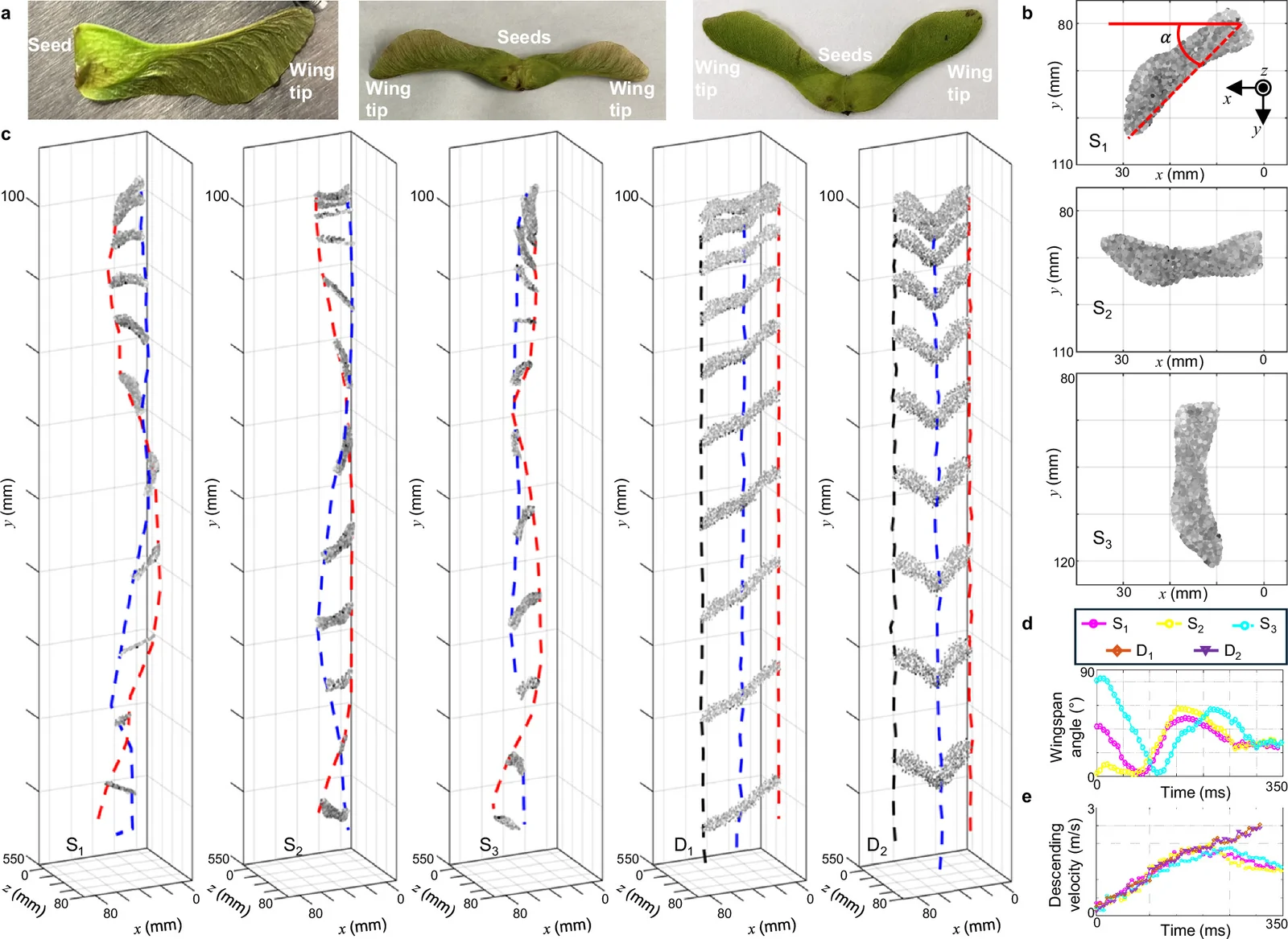
C-BLIP of falling maple samaras. **a** 2D photos of three tested maple samaras: single-winged samara (left), flat dual-winged samara (middle), and angled dual-winged samara (right). **b** 3D reconstructions of the single-winged samara with initial wingspan angles (α) of 45° (top), 0° (middle), and 90° (bottom). **c** Representative 3D images showing the falling dynamics of the single-winged samaras with initial conditions specified in (**b**), the flat dual-winged samara (D_1_), and the angled dual-winged samara (D_2_). Dashed lines: 3D trajectories of the seed (blue) and wing tips (red and black). **d** Evolution of the wingspan angles of the single-winged samaras. **e** Descending speeds of five cases shown in (**c**).

To apply C-BLIP to bionics, we recorded freely falling Norway maple (*Acer platanoides*) samaras^54^ using the intensity-3D-imaging mode with the DMD’s refresh rate at 25 kHz. In this mode, the depth resolution was quantified to be 0.93 ± 0.14 mm. Fig. 4a presents one single-winged samara as well as two dual-winged samaras with flat and angled wingspans. For the single‑winged samara, we performed three drop trials with distinct wingspan angles—defined as the angle between the seed edge-wing tip vector and the horizontal plane^55^ (x-z in this experiment) — of α = 45°, 0°, and 90° (labeled as S_1_–S_3_ in Fig. 4b). For comparison, both the flat (labeled as D_1_) and angled (labeled as D_2_) dual-winged samaras were dropped with their leading edge facing downward. Fig. 4c shows 11 representative C-BLIP’s 3D reconstructions for single-winged samaras and 10 for dual-winged samaras at 32 ms time intervals, revealing different initial transient descents as the samara adjusted its orientation (full evolutions shown in Supplementary Movie 4). To characterize the falling dynamics, we tracked the 3D trajectories of the seed edge and wing tip for each case, displayed as the dashed lines in Fig. 4c. For the single-winged samara, despite different initial orientations and variable delays before autorotation onset, all three trials (i.e., S_1_–S_3_) converged to a helicopter-style autorotation centered on the seed edge at the end of the observation period. In contrast, neither dual-winged morphology (i.e., D_1_ and D_2_) achieved autorotation. Rather, their descent was dominated by translation punctuated by small wiggling motions. Both observations are consistent with the literature^56,57^.

To further quantify the autorotation kinematics of the single-winged samaras, we tracked their wingspan angle under the three different initial orientations (i.e., S_1_–S_3_). As shown in Fig. 4d, the evolution of this angle exhibits a consistent sequence across all three conditions: regardless of its initial orientation, the samara automatically adjusts its orientation, as gravity pulls the seed edge downward and air begins to interact with the wing. This process results in a monotonically increasing wingspan angle, reaching a peak between α = 50° and 60°. During this phase, the samara’s asymmetric mass and geometry generate a torque, producing a small rotational moment that enables the wing to generate aerodynamic lift. This lift enhances spin, as indicated by the subsequent decrease in the wingspan angle. Finally, gyroscopic and aerodynamic forces stabilize the axis of rotation, which leads the samara into a stable autorotation mode. In our experiments, all specimens converged to a similar wingspan angle of α = 27.9 ± 3.1°.

The different fall dynamics of the single- and dual-winged samaras are also reflected in their descending speeds, as shown in Fig. 4e. Each samara initially accelerates under gravity until approximately 150 ms. After this time point, the dual-winged samaras continue accelerating unabated, reflecting their gravity-dominated descent. By contrast, the single-winged samaras reach their maximum speed in tandem with the peak wingspan angle and subsequently decelerate to ~1300 mm/s—closely matching previously reported values^54,55^. As an example, the evolution of the falling dynamics of S_1_ between 250 and 350 ms is displayed in Supplementary Movie 5.

### Intensity 3D imaging of encrypted ultrasound signals at 150 kfps

**Fig. 5:**
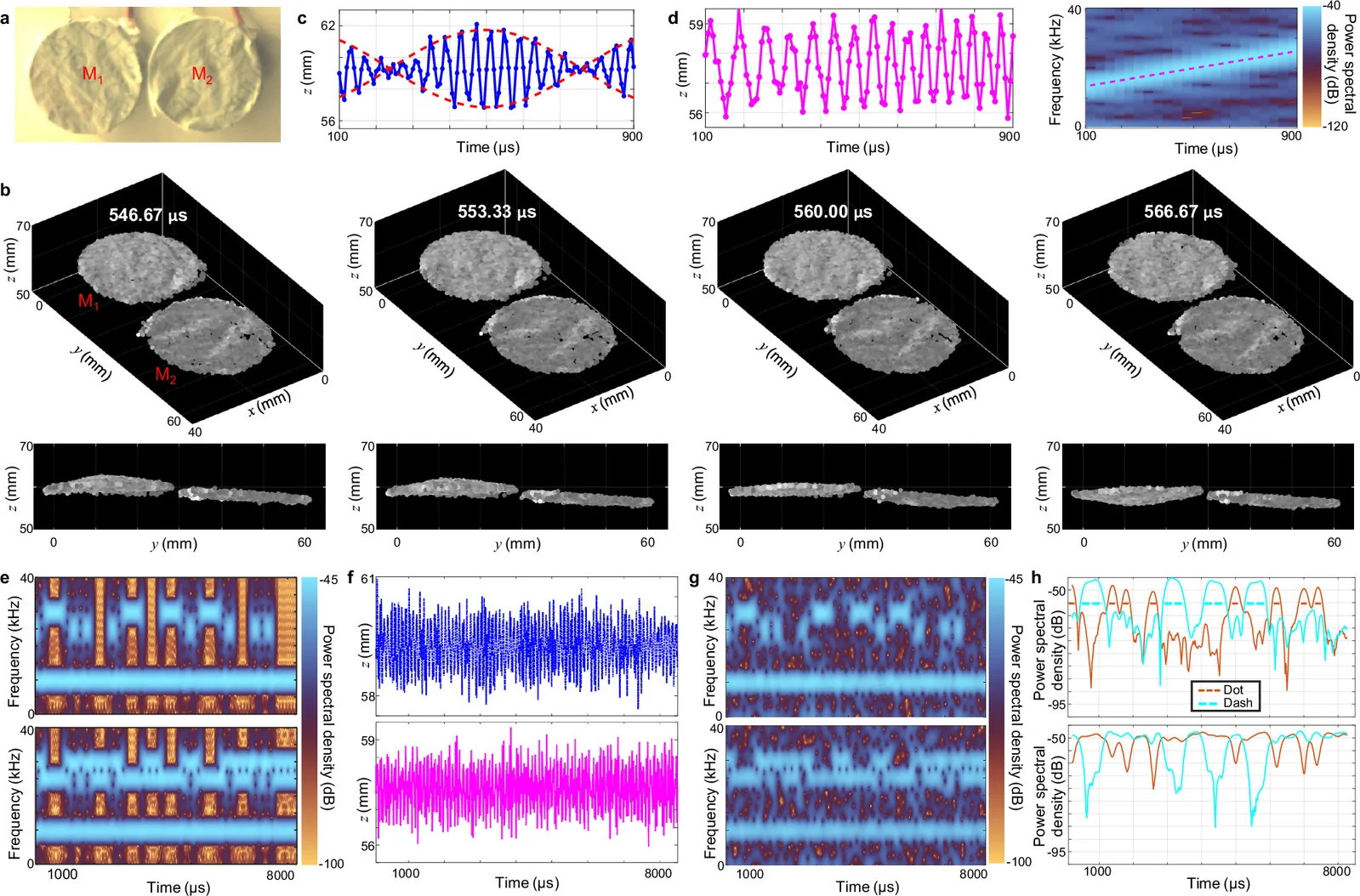
C-BLIP of ultrasound audio encryption. **a** 2D photograph of two speakers marked as M_1_ and M_2_. **b** Oblique view (top row) and side view (bottom row) of four representative 3D reconstructions of the speakers. **c** Time history of the mean depth of M_1_’s central region. Red dashed lines: fitted beat-frequency envelope of the measurement. **d** Time history of the mean depth of M_2_’s central region (left) and the corresponding power spectrogram (right). Red dashed line: ground truth frequency content of the input signal. **e** Power spectrograms of the encoded Morse signals input to M_1_ (top) and M_2_ (bottom). **f–h** Reconstructed time histories of the mean depths (**f**), the power spectrograms (**g**), and the line profiles of the power spectrograms (**h**) at 25 kHz (orange) and 30 kHz (aqua) for M_1_ (top) and M_2_ (bottom).

To apply C-BLIP to information security, we mapped the membrane vibrations of two adjacent loudspeakers (Meetoot KS-3840A, labeled as M_1_ and M_2_) broadcasting ultrasound signals (Fig. 5a). C-BLIP was operated in the intensity-3D-imaging mode with the 50-kHz DMD’s refresh rate by using an encoding ROI of 80 × 1140 pixels, with a depth resolution of 1.10 ± 0.18 mm. To verify C-BLIP’s performance, we applied distinct waveforms to the two loudspeakers: M_1_ was driven by a dual‑tone beat signal consisting of 24 and 26 kHz sinusoidal waves, creating a 2 kHz beat frequency, while M_2_ received a linearly chirped signal from 10 to 50 kHz (full evolution shown in Supplementary Movie 6). Fig. 5b displays four representative 3D reconstructions of both membranes at 6.67 µs intervals, each shown from two perspectives: an oblique view illustrating the membrane surface geometry and a side view highlighting the depth information and visualizing the membrane oscillations. Fig. 5c plots the mean depth of the central region of M_1_ (5 × 5 mm in size) over time, overlaid with the fitted curve indicating the beat frequency of the envelope being 1.92 kHz, closely matching the input signal. Within this envelope, C-BLIP also clearly resolved the underlying high-frequency oscillations. Moreover, Fig. 5d presents the mean depth of the central region of M_2_ (5 × 5 mm in size), along with its corresponding spectrogram computed via a short-time Fourier transform using a 64-sample window length and a 4-sample step. The result closely matches the ground truth.

To further explore C-BLIP’s utility in audio encryption^58^, we designed a dual‑speaker encoding scheme that embedded a four-letter message (“LACI”) in Morse code into ultrasound bands (i.e., 25 kHz and 30 kHz for the encoded dots and dashes) while masking it with a continuous 10-kHz audible background noise. Following International Morse timing rules^59^, we defined that a dot corresponds to one unit (200 µs), a dash to three units (600 µs), intra-symbol gaps to one unit (200 µs), and inter-letter gaps to three units (600 µs). As shown in Fig. 5e, M_1_ carries the message of “LACI” encrypted by Morse code—L(**·**^**‑**^**··**), A(**·**^**‑**^), C(^**‑**^**·**^**‑**^**·**), and I(**··**)—and embeds it in the spectrogram. Meanwhile, serving as a decoy, M_2_ plays a complementary signal that fills the unused 25 kHz and 30 kHz intervals. These spectral patterns were then converted into time‑domain amplitude waveforms via inverse spectral synthesis and delivered to both speakers through amplifiers. Detailed encryption procedures and system operation are provided in Supplementary Note 7 and Supplementary Fig. 5. This scheme implements a double-encryption mechanism: in addition to Morse coding, an ultrasound transducer cannot isolate either component from the two complementary signals, effectively preventing unauthorized access to the hidden Morse code. In contrast, leveraging its spatially resolved vibration detection, C-BLIP reveals the correct ultrasound signal of each speaker. The full evolution of this event is shown in Supplementary Movie 7. The mean depth traces and spectrograms for M_1_ and M_2_ in Fig. 5f, g show clear bursts at 25 kHz and 30 kHz, respectively, but with distinct temporal locations. Finally, we extracted line profiles at the two designed frequencies and applied a −55 dB threshold, successfully recovering the exact Morse sequence from M_1_ (Fig. 5h). On the contrary, M_2_’s profiles contain no discernible coded information.

### Intensity 3D imaging of ultrasound-encoded audio recovery at 150 kfps

**Fig. 6:**
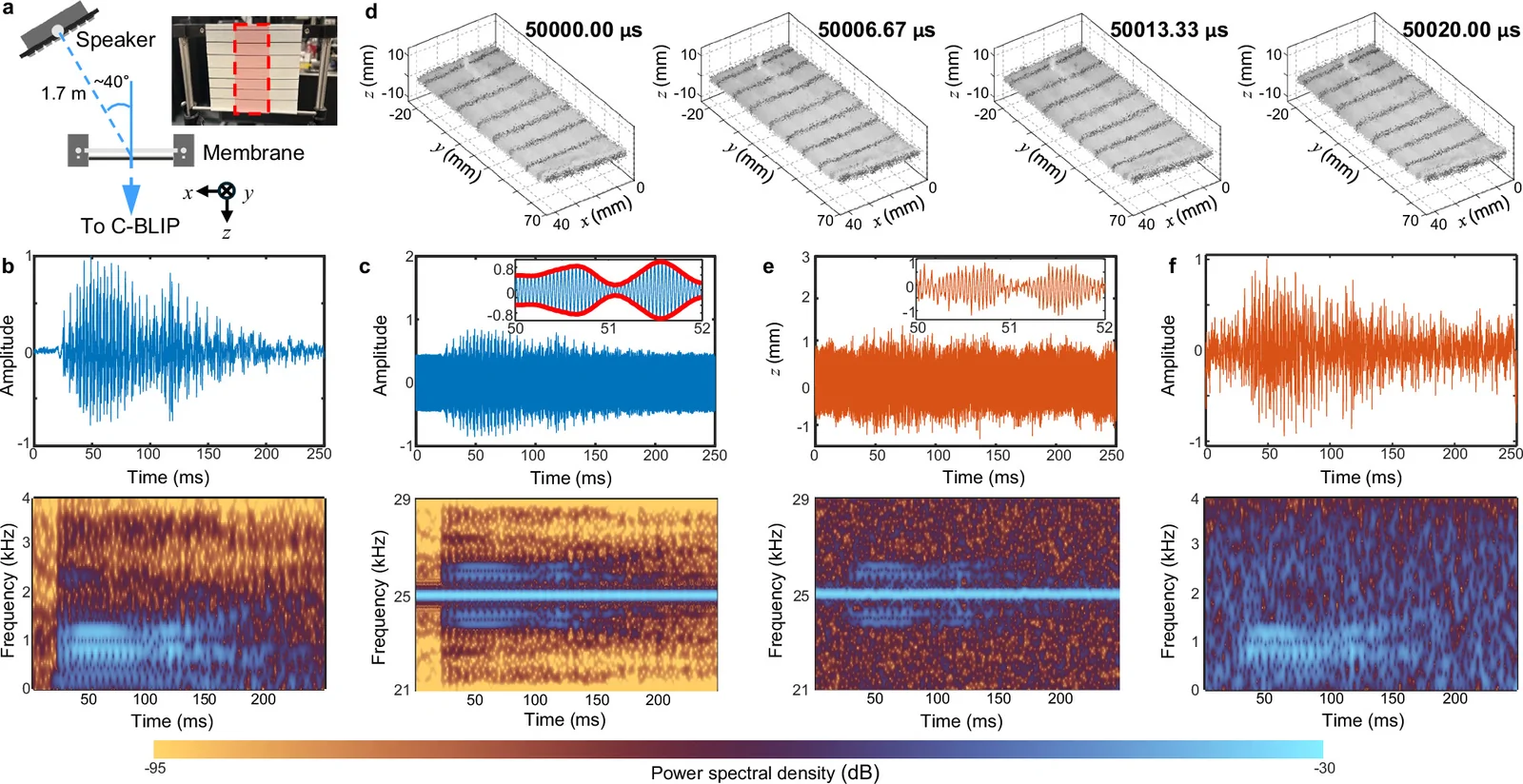
C-BLIP of ultrasound-encoded audio recovery. **a** Schematic of the experimental setup. Inset: photo of the curtain-mimicking membrane. C-BLIP’s field of view is marked by the red-dashed box. **b** Baseband waveform of the spoken word “Hi” (top) and its power spectrogram (bottom). **c** Audio waveform modulated by a 25-kHz carrier frequency (top) and its power spectrogram (bottom). Inset: zoomed-in view of the carrier oscillation under its envelope (red) over 50–52 ms. **d** Four representative 3D reconstructions of the membrane at 6.67-µs intervals, shown in oblique view. **e** Mean depth of the membrane’s central region (top) and its power spectrogram (bottom) over time. Inset: zoomed-in view of the reconstructed depth trace over 50–52 ms. **f** Recovered audio waveform (top) and its power spectrogram (bottom).

Beyond protecting information, C-BLIP can also recover audio signals from the ultrasound-induced motion of lightweight structures. As shown in Fig. 6a, we mapped the vibrations of a thin plastic suspended membrane, placed 1.7 meters away from a loudspeaker (DIY, DS100A), with an incident angle of ~40° to the membrane’s normal. The loudspeaker broadcast the spoken word “Hi”, whose baseband waveform and its corresponding spectrogram are shown in Fig. 6b. This audio was encoded onto a 25-kHz carrier by amplitude modulation (Fig. 6c). This modulated waveform was then fed to the loudspeaker, whose output drove the membrane into vibration. To mimic a curtain, the membrane was fixed along its top edge and weighted with a bar along its bottom edge, leaving it free to vibrate under the acoustic drive (Fig. 6a inset). C-BLIP was operated in the intensity-3D-imaging mode at 150 kfps. Additional experimental details on acoustic spectrum analysis and amplitude modulation are included in Supplementary Note 8.

The reconstructed dynamic scene is shown in Supplementary Movie 8. Four representative frames at 6.67-µs intervals are displayed in Fig. 6d. To quantify the acoustically induced vibrations of the membrane surface, we extracted the mean depth of the central region of the membrane (5 × 5 mm in size) over time (Fig. 6e, top). The spectrogram of this depth trace (Fig. 6e, bottom) shows the 25-kHz carrier and its surrounding sideband structure. The speech waveform was recovered by single-sideband demodulation (Fig. 6f, top; detailed in Supplementary Note 8), yielding a normalized root mean square error (NRMSE) of 13.33% to the ground truth. The corresponding power spectrogram (Fig. 6f, bottom) resembles the ground truth (Fig. 6b, bottom), which is further illustrated by the NRMSE of 12.66% and 16.01% between the two spectrograms at the dominant speech bands of 0.71 kHz and 1.18 kHz. The recovered waveform was rendered back into an audio file that reproduces the spoken “Hi” (Supplementary Movie 8). Together, these results confirm that speech can be recovered from an encoded ultrasound signal by C-BLIP.

## Discussion

We have developed C-BLIP as a versatile approach for multiscale, ultrahigh-speed, continuous-streaming 3D surface imaging. We exploited the DMD’s encoding ROI to increase its refresh rate to up to 50 kHz. We also designed and fabricated a projection lens with chromatically limited spatial bandwidth. Together with the finite spectral bandwidth of incoherent LEDs, C-BLIP’s projection module generates a precise grayscale sinusoidal fringe at the object plane from a single DMD binary mask without spatial filtering or defocusing. Up to 12 fringe images with varied periods, orientations, and colors are multiplexed onto a color camera for the ensuing data processing and 3D image reconstruction. We demonstrated C-BLIP’s color 3D imaging at 25 kfps by operating the DMD at 25 kHz while simultaneously turning on all three LEDs. We also used C-BLIP for intensity 3D imaging at 75 kfps and 150 kfps by controlling the DMD’s encoding ROI and sequentially illuminating each LED. C-BLIP overcomes the limitations of existing ultrahigh-speed SS-FPP systems. Compared to the methods based on high-speed projection, C-BLIP demonstrates the highest projection rate of 50 kHz by using the encoding ROI. Compared to approaches leveraging advanced high-speed detection, C-BLIP employs multiplexing on a machine-vision camera, which not only reduces cost considerably but, more importantly, enables continuous streaming. Finally, C-BLIP taps into the potential of color LEDs by intentionally accounting for their finite spectral bandwidth in the optical design. The resulting chromatically band-limited projection enables high-precision fringe generation under incoherent illumination, without requiring a physical low-pass filter. Altogether, C-BLIP exhibits itself as a flexible, eye-safe, economical, ultrahigh-speed 3D surface imaging tool with broad applicability.

Future research will further improve C-BLIP’s imaging capability. Complex LED illumination schemes could be teamed up with learning-based spectral unmixing techniques to increase the number of spectral channels^60^. Video-frame interpolation could recover finer temporal features between the two adjacent captured frames^61–63^. Both approaches could enhance C-BLIP’s imaging speed without changing the current C-BLIP architecture. Further improvement could be accomplished via a hardware upgrade. For example, limited by the DMD’s encoding ROI, C-BLIP’s field of view (FOV) is currently restricted to 3.5 cm × 24.9 cm in the 150-kfps intensity-3D-imaging mode. In the future, a tiled-DMD array might expand the effective FOV without sacrificing resolution^64^. For the camera to match this FOV, C-BLIP’s multiplexing capacity will be further expanded by filling the usable Fourier band with more carrier frequencies while synthetically considering the imaging content of the 3D object and signal-to-noise ratios in the measurements. Moreover, LEDs with different emission wavelengths can be used with a snapshot multi-spectral camera to increase the imaging speed^65^. LEDs may overheat during extended use, which can shift their spectra and thereby alter the wavelength-dependent MTF and potentially damage the emitters. Future designs can incorporate enhanced heat sinking or active cooling. Alternatively, LEDs with wavelengths in the near-infrared or ultraviolet range, which exhibit lower thermal sensitivity, can be used for better heat management^66–68^. Finally, on the data‑processing side, real‑time demultiplexing and FTP reconstruction accelerated by a graphics processing unit would enable live 3D visualization for feedback‑controlled experiments.

As a generic platform, C-BLIP holds promise for future applications. C-BLIP of high-speed turbofan collision tests (Fig. 3) demonstrates its potential for non-contact, ultrahigh-speed diagnostics and fatigue testing of rotating machinery^69,70^. Its implementation into aerodynamics studies of descending maple samaras (Fig. 4) provides quantitative data for bio-inspired robotics and ecological seed-dispersal modeling^71^. C-BLIP’s video recording of ultrasound-induced vibration 3D visualization (Fig. 5) sheds light on non-invasive vibration diagnostics^72^ and secure underwater or structural communications^73^. Finally, C-BLIP’s audio recovery from vibrations of a lightweight membrane (Fig. 6) shows its potential for applications in remote acoustic sensing and surveillance^74^. Beyond these demonstrations, C-BLIP is readily applicable to real-time 3D measurement of microfluidic droplet formation and breakup^75^, dynamic fracture and crack-tip mapping in high-rate material testing^76^, and in-situ monitoring of melt-pool dynamics in laser-based additive manufacturing^77^.

## Methods

### Design of the chromatically band-limited projection lens

The projection lens, designed in-house using Zemax, aims to provide a compact and economical solution for projecting grayscale fringe patterns without the need for a physical low-pass filter or optical defocusing. Two specific objectives guided the lens design. First, the lens should have an effective focal length of 6 mm to enable a high magnification ratio. Second, the MTF should be wavelength-dependent to produce a smooth-edged low-pass filter by leveraging the spectrum bandwidth of incoherent LEDs.

Toward this goal, the projection lens was designed through the following major steps. We began with an in-catalog projection lens model^78^. After the ideal prescription was established, each element was substituted with the closest available off-the-shelf singlet lens to keep the design affordable and avoid custom lens fabrication.

Subsequently, the lens was optimized for the central wavelengths of the used LEDs (e.g., 460 nm, 527 nm, and 620 nm). Merit functions were defined to preserve the 6-mm effective focal length while simultaneously specifying MTF targets for optimized wavelengths to be ~0.5 within the spatial frequency range bounded by the shortest periods accommodated by the DMD pixel size (d = 7.6 µm; ${f}_{2d}$ = 65.8 cycles/mm) and its pitch size (p=10.8 µm; ${f}_{2p}$ = 46.3 cycles/mm). As demonstrated in Fig. 1c, d, after optimization, the lens design exhibits the desired MTF characteristics for all three wavelengths. Outside these wavelengths, the MTF decreases and cuts off toward the individual DMD pixel limit.

Next, broadband MTFs were computed in Zemax. In particular, the monochromatic MTF at each wavelength was weighted by the associated light intensity and then summed to obtain the broadband MTFs. As shown in Fig. 1d, the broadband MTFs roll off more steeply than the single-wavelength counterparts, approaching a cutoff near ${f}_{2d}$. Meanwhile, for the spatial frequencies of the fringes residing in the low-frequency domain, the broadband and optimized monochromatic MTFs closely coincide, maintaining high MTF values close to one. This result shows that the synergy of this lens design and the broadband LED spectra can produce a smooth-edged low-pass filter.

Following this design process, several configurations were evaluated by using lenses from various vendors. For each configuration, the broadband MTFs and the optimized single-wavelength MTFs were compared over the range bounded by ${f}_{2p}$ and ${f}_{2d}$. The configuration that maximized the drop-off between those two curves was selected, as shown in Supplementary Fig. 1a and Supplementary Table 1. The simulation of the produced sinusoidal fringes and their accuracy analysis are shown in Fig. 1, f. The extracted radial profiles closely match the ground truth sinusoidal fringes with NRMSEs below 5%. The final configuration achieved an effective focal length of 6.0 mm and an F-number of 2.8, with an overall track length of 148.7 mm. This projection lens was fabricated by mounting the individual singlet lenses in a 3D-printed barrel with precision spacers to set the inter-element distances (Supplementary Fig. 1b).

### Image reconstruction of C-BLIP

The data processing pipeline is outlined in Supplementary Fig. 3a with an example illustrated in Supplementary Fig. 3b. We first describe the procedure of intensity-3D-imaging mode. C-BLIP first demultiplexes the multi-fringe encoded images from individual color channel images, and then implements FTP for phase analysis in its 3D reconstruction^5,19^. Since Fringes 1–4 are projected onto the object sequentially within one camera exposure, the separated single color channel image is the superposition of four intensity fringe images. The overall intensity profile of a camera pixel (${u}_{{{{\rm{c}}}}}$, ${v}_{{{{\rm{c}}}}}$) on the single-channel image can be expressed as

1 $${I}^{s}\left({u}_{{{{\rm{c}}}}},\,{v}_{{{{\rm{c}}}}}\right)={\sum}_{i}\left\{{I}_{{{{\rm{b}}}}}^{s,i}\left({u}_{{{{\rm{c}}}}},\,{v}_{{{{\rm{c}}}}}\right)+{I}_{{{{\rm{v}}}}}^{s,i}\left({u}_{{{{\rm{c}}}}},\,{v}_{{{{\rm{c}}}}}\right)\cos \left[2\pi {M}_{i}\left({u}_{{{{\rm{c}}}}},\,{v}_{{{{\rm{c}}}}}\right)+{\varphi }^{s,i}\left({u}_{{{{\rm{c}}}}},\,{v}_{{{{\rm{c}}}}}\right)\right]\right\}$$

Here, s=[R, G, B], ${{M}_{i}\left({u}_{{{{\rm{c}}}}},{v}_{{{{\rm{c}}}}}\right)=u}_{{{{\rm{c}}}}}{f}_{i}\cos {\theta }_{i}+{v}_{{{{\rm{c}}}}}{f}_{i}\sin {\theta }_{i}$ (i=1–4), ${f}_{i}$ is the spatial frequency of fringes, and ${\theta }_{{i}}$ represents fringe orientation. ${I}_{{{{\rm{b}}}}}^{s,i}({u}_{{{{\rm{c}}}}},\,{v}_{{{{\rm{c}}}}})$, ${I}_{{{{\rm{v}}}}}^{s,i}({u}_{{{{\rm{c}}}}},\,{v}_{{{{\rm{c}}}}})$, and ${\varphi }^{s,i}\left({u}_{{{{\rm{c}}}}},\,{v}_{{{{\rm{c}}}}}\right)$ represent the background, intensity variation, and depth-dependent phase, respectively.

The fringe modulation term in Eq. (1) can be rewritten in its complex form as

2 $$\cos \left[2\pi {M}_{i}\left({u}_{{{{\rm{c}}}}},\,{v}_{{{{\rm{c}}}}}\right)+{\varphi }^{s,i}\left({u}_{{{{\rm{c}}}}},\,{v}_{{{{\rm{c}}}}}\right)\right]=\frac{1}{2}\,\left\{{e \, }^{j\left[2\pi {M}_{i}\left({u}_{{{{\rm{c}}}}},{v}_{{{{\rm{c}}}}}\right)+{\varphi }^{s,i}\left({u}_{{{{\rm{c}}}}},{v}_{{{{\rm{c}}}}}\right)\right]}+{e}^{-j\left[2\pi {M}_{i}\left({u}_{{{{\rm{c}}}}},{v}_{{{{\rm{c}}}}}\right)+{\varphi }^{s,i}\left({u}_{{{{\rm{c}}}}},{v}_{{{{\rm{c}}}}}\right)\right]}\right\}$$

Applying a 2D Fourier transform of Eq. (1) gives

3 $${\tilde{I}}^{s}\left({f}_{x},\,{f}_{y}\right)={\sum}_{i}\left[{\tilde{I}}_{{{{\rm{b}}}}}^{s,i}\left({f}_{x},\,{f}_{y}\right)+{\tilde{I}}_{{{{\rm{f}}}}}^{s,i}\left({f}_{x}-{f}_{i}\cos {\theta }_{i},\,{f}_{y}-{f}_{i}\sin {\theta }_{i}\right)+{\tilde{I}}_{{{{\rm{f}}}}}^{s,i}\left({f}_{x}+{f}_{i}\cos {\theta }_{i},\,{f}_{y}+{f}_{i}\sin {\theta }_{i}\right)\right]$$

Here, $\left({f}_{x},\,{f}_{y}\right)$ are spatial frequency coordinates. ${\widetilde{I}}_{{{{\rm{b}}}}}^{s,i}\left({f}_{x},\,{f}_{y}\right)$ and ${\widetilde{I}}_{{{{\rm{f}}}}}^{s,i}\left({f}_{x}-{f}_{i}\cos {\theta }_{i},\,{f}_{y}-{f}_{i}\sin {\theta }_{i}\right)$ are the Fourier transforms of ${I}_{{{{\rm{b}}}}}^{s,i}\left({u}_{{{{\rm{c}}}}},\,{v}_{{{{\rm{c}}}}}\right)$ and $\frac{1}{2}\cdot {I}_{{{{\rm{v}}}}}^{s,i}\left({u}_{{{{\rm{c}}}}},\,{v}_{{{{\rm{c}}}}}\right){e}^{j[2\pi {M}_{i}\left({u}_{{{{\rm{c}}}}},{v}_{{{{\rm{c}}}}}\right)+{\varphi }^{s,i}\left({u}_{{{{\rm{c}}}}},{v}_{{{{\rm{c}}}}}\right)]}$, respectively.

For demodulation, ${\widetilde{I}}^{s}\left({f}_{x},\,{f}_{y}\right)$ is band-pass filtered by a Hann window centered at $({f}_{i}\cos {\theta }_{i},\,{f}_{i}\sin {\theta }_{i})$ to suppress the dc component and all other lobes, shifted to the origin, and inverse Fourier transformed to obtain

4 $$\hat{I}^{s,i}({u}_{{\mathrm{c}}},{v}_{{\mathrm{c}}})=\frac{1}{2} \cdot {I}_{{{{\rm{V}}}}}^{s,i}({u}_{{\mathrm{c}}},{v}_{{\mathrm{c}}}){e \, }^{{j\varphi }^{{s,i}_{({u}_{{\mathrm{c}}},{v}_{{\mathrm{c}}})}}}$$

The depth-dependent phase is then extracted as

5 $${\varphi }^{s,i}\left({u}_{{{{\rm{c}}}}},\,{v}_{{{{\rm{c}}}}}\right)={\tan }^{-1}\left\{\frac{{\mathfrak{I}}\left[\hat{I}^{s,i}\left({u}_{{{{\rm{c}}}}},\,{v}_{{{{\rm{c}}}}}\right)\right]}{{\mathfrak{R}}\left[\hat{I}^{s,i}\left({u}_{{{{\rm{c}}}}},\,{v}_{{{{\rm{c}}}}}\right)\right]}\right\}$$

where ${\mathfrak{I}}{\mathfrak{[}}{\mathfrak{]}}$ and ${\mathfrak{R}}{\mathfrak{[}}{\mathfrak{]}}$ denote the imaginary and real parts of a complex variable, respectively. Equation (5) calculates a phase value ranging from $(-\,\pi,\,+\,\pi ]$, which refers to the wrapped phase. Considering that the three LEDs illuminate sequentially in the intensity-3D-imaging mode (Supplementary Fig. 3c), each ${I}_{{{{\rm{v}}}}}^{s,i}\left({u}_{{{{\rm{c}}}}},\,{v}_{{{{\rm{c}}}}}\right)$ and ${\varphi }^{s,i}\left({u}_{{{{\rm{c}}}}},\,{v}_{{{{\rm{c}}}}}\right)$ can be associated with a specific time point. The color information in ${I}_{{{{\rm{v}}}}}^{s,i}\left({u}_{{{{\rm{c}}}}},\,{v}_{{{{\rm{c}}}}}\right)$ is also discarded.

Then, the continuous phase is recovered by the 2D Goldstein branch cut phase unwrapping algorithm^79^ as

6 $${\widetilde{\varphi }}^{s,i}\left({u}_{{{{\rm{c}}}}},\,{v}_{{{{\rm{c}}}}}\right)={\varphi }^{s,i}\left({u}_{{{{\rm{c}}}}},\,{v}_{{{{\rm{c}}}}}\right)+2\pi \cdot {k}^{s,i}\left({u}_{{{{\rm{c}}}}},\,{v}_{{{{\rm{c}}}}}\right)$$

where ${k}^{s,i}\left({u}_{{{{\rm{c}}}}},\,{v}_{{{{\rm{c}}}}}\right)$ is the integer fringe-order value. For each fringe orientation ${\theta }_{i}$, a pre-projected centerline, which has a finite width and is parallel to the fringes, marks the projector center, where the absolute phase is set to zero. For each camera pixel $\left({u}_{{{{\rm{c}}}}},\,{v}_{{{{\rm{c}}}}}\right)$, a line through $\left({u}_{{{{\rm{c}}}}},\,{v}_{{{{\rm{c}}}}}\right)$ along the phase-gradient direction determined by ${\theta }_{i}$ intersects the imaged centerline ${{{{\mathscr{L}}}}}_{i}$. The unwrapped absolute phase is calculated by

7 $${\hat{\varphi }}^{s,i}\left({u}_{{{{\rm{c}}}}},\,{v}_{{{{\rm{c}}}}}\right)={\widetilde{\varphi }}^{s,i}\left({u}_{{{{\rm{c}}}}},\,{v}_{{{{\rm{c}}}}}\right)-\overline{{\widetilde{\varphi }}^{s,i}({u}_{{{{\rm{c|}}}}{{{{\mathscr{L}}}}}_{i}},\,{v}_{{{{\rm{c|}}}}{{{{\mathscr{L}}}}}_{i}})}$$

where the reference phase $\overline{{\widetilde{\varphi }}^{s,i}({u}_{{{{\rm{c|}}}}{{{{\mathscr{L}}}}}_{i}},\,{v}_{{{{\rm{c|}}}}{{{{\mathscr{L}}}}}_{i}})}$ is defined as the mean of the unwrapped phases of the intersected centerline pixels, denoted by $({u}_{{{{\rm{c|}}}}{{{{\mathscr{L}}}}}_{i}},\,{v}_{{{{\rm{c|}}}}{{{{\mathscr{L}}}}}_{i}})$. Thus, the projector phase line can be obtained by ${u}_{{{{\rm{p}}}}}\cos {\theta }_{i}+{v}_{{{{\rm{p}}}}}\sin {\theta }_{i}={\hat{\varphi }}^{s,i}\left({u}_{{{{\rm{c}}}}},\,{v}_{{{{\rm{c}}}}}\right)/(2\pi {f}_{i})$. Finally, by combining with the system calibration matrices $$\left[\begin{array}{ccc}{a}_{11} & \cdots & {a}_{14}\\ \vdots & \ddots & \vdots \\ {a}_{31} & \cdots & {a}_{34}\end{array}\right]$$ and $$\left[\begin{array}{ccc}{b}_{11} & \cdots & {b}_{14}\\ \vdots & \ddots & \vdots \\ {b}_{31} & \cdots & {b}_{34}\end{array}\right]$$ (Supplementary Note 4), the world coordinates of a 3D point (i.e., [*x, y, z*]^*T*^) can be calculated as^5^

8 $$	\left[\begin{array}{c}x\\ y\\ z\end{array}\right]={\left[\begin{array}{ccc}{a}_{11}-{u}_{{{{\rm{c}}}}}{a}_{31} & {a}_{12}-{u}_{{{{\rm{c}}}}}{a}_{32} & {a}_{13}-{u}_{{{{\rm{c}}}}}{a}_{33}\\ {a}_{21}-{v}_{{{{\rm{c}}}}}{a}_{31} & {a}_{22}-{v}_{{{{\rm{c}}}}}{a}_{32} & {a}_{23}-{v}_{{{{\rm{c}}}}}{a}_{33}\\ {b}_{11}\cos {\theta }_{i}+{b}_{21}\sin {\theta }_{i}-{b}_{31}\\ \left({u}_{{{{\rm{p}}}}}\cos {\theta }_{i}+{v}_{{{{\rm{p}}}}}\sin {\theta }_{i}\right) & {b}_{12}\cos {\theta }_{i}+{b}_{22}\sin {\theta }_{i}-{b}_{32}\left({u}_{{{{\rm{p}}}}}\cos {\theta }_{i}+{v}_{{{{\rm{p}}}}}\sin {\theta }_{i}\right) & {b}_{13}\cos {\theta }_{i}+{b}_{23}\sin {\theta }_{i}-{b}_{33}\left({u}_{{{{\rm{p}}}}}\cos {\theta }_{i}+{v}_{{{{\rm{p}}}}}\sin {\theta }_{i}\right)\end{array}\right]}^{{{-}}{{{\bf{1}}}}}\\ 	 \left[\begin{array}{c}{u}_{{{{\rm{c}}}}}{a}_{34}-{a}_{14}\\ \,{v}_{{{{\rm{c}}}}}{a}_{34}-{a}_{24}\\ {b}_{34}\left({u}_{{{{\rm{p}}}}}\cos {\theta }_{i}+{v}_{{{{\rm{p}}}}}\sin {\theta }_{i}\right)-{b}_{14}\cos {\theta }_{i}-{b}_{24}\sin {\theta }_{i}\end{array}\right]$$

In this way, a single multiplexed image outputs a 12-frame 3D video.

In the color-3D-imaging mode, the data processing largely follows that of the intensity-3D-imaging mode with two key differences. First, the three LEDs illuminate simultaneously (Supplementary Fig. 3d). Thus, for a given fringe, the demultiplexed R/G/B images encode the scene at the same time, and the phase map is therefore color-channel-invariant. A single channel’s phase map, ${\hat{\varphi }}^{s,i}$ by default, is used for triangulation to produce one 3D reconstruction. Second, the per-channel amplitudes ${I}_{{{{\rm{v}}}}}^{{{{\rm{R}}}},i}\left({u}_{{{{\rm{c}}}}},\,{v}_{{{{\rm{c}}}}}\right)$, ${I}_{{{{\rm{v}}}}}^{{{{\rm{G}}}},i}\left({u}_{{{{\rm{c}}}}},\,{v}_{{{{\rm{c}}}}}\right)$, and ${I}_{{{{\rm{v}}}}}^{{{{\rm{B}}}},i}\left({u}_{{{{\rm{c}}}}},\,{v}_{{{{\rm{c}}}}}\right)$ are combined to form one RGB image, which is mapped onto the 3D reconstruction. In this way, a single multiplexed image outputs a 4-frame color 3D video.

## Supplementary information

Supplementary Information

Description of Additional Supplementary Files

Supplementary Movie 1

Supplementary Movie 2

Supplementary Movie 3

Supplementary Movie 4

Supplementary Movie 5

Supplementary Movie 6

Supplementary Movie 7

Supplementary Movie 8

Transparent Peer Review file

## Data Availability

All data needed to evaluate the findings of this study are present in the paper and Supplementary Information. Representative raw data are provided with the software package (https://doi.org/10.5281/zenodo.22073518).

## Code availability

A MATLAB software package is available at https://doi.org/10.5281/zenodo.22073518.
