# Supplementary material for: Ultrahigh-speed chromatically band-limited illumination profilometry
Source: Nat Commun. 2026 Oct 12;17:11567. doi: 10.1038/s41467-026-78478-w (PMC13748529; doi:10.1038/s41467-026-78478-w)
Supplement: Supplementary file 1 [file 41467_2026_78478_MOESM11_ESM.pdf]

nature portfolio

Peer Review File

Ultrahigh-speed chromatically band-limited illumination profilometry

Corresponding Author: Professor Jinyang Liang

This file contains all reviewer reports in order by version, followed by all author rebuttals in order by version.

Version 0:

Reviewer comments:

Reviewer #1

(Remarks to the Author)
The authors presented a novel three-dimensional shape measurement technique named chromatically band-limited illumination profilometry. The method aims to overcome a limitation of conventional 3D profilometry, namely, measurement speed. By combining RGB LED illumination with a high-speed DMD for fringe generation and a carefully designed projection lens system, the authors project fringe patterns onto the object and reconstruct 3D information from camera-captured images. To highlight the capability of the proposed method, the authors presented imaging and analysis demonstrations of fast dynamics such as a turbofan blade-projectile collision and maple-samara fall aerodynamics.

While binary patterns generated by DMDs are generally inferior in spatial quality compared to those produced by liquid-crystal SLMs, DMDs offer significantly higher switching speeds. In this work, the authors cleverly introduce an intentional spatial-frequency limitation in the projection optics to improve fringe quality, effectively resolving this trade-off. This design strategy is particularly compelling. In addition, the system description provided in the Supplementary Information is thorough and detailed. It presents a technical platform that is likely to stimulate derivative research and practical extensions.

Overall, this study is interesting and significant. This manuscript presents sufficient analysis and experimental validation to substantiate the proposed method. Therefore, I recommend publishing this work in Nature Communications, provided that the following comments are addressed.

Major comments:
The technical contribution of this work is clear and compelling. To further strengthen the manuscript, I suggest clarifying the core innovation more explicitly in the main text.
After introducing the system configuration in the Results section, the manuscript proceeds directly to multiple demonstrations. It may be beneficial to more explicitly highlight the principal advantages of the proposed method before presenting these demonstrations, as this would help guide readers on what aspects to focus on when interpreting the subsequent imaging results. As high-speed operation is emphasized in the Abstract and Introduction, this clarification could particularly include concise quantitative statements regarding the frame rates and related parameters, as well as a clear explanation of how the proposed design overcomes the bottlenecks of conventional methods.
In addition, a brief summary of the projection lens design principles in the main text could further enhance the readability and impact of the paper.

Minor Comment:
Regarding system resolution, the Results section refers readers to Supplementary Note 4. It would be helpful to include values under specific conditions directly in this paragraph, as this would facilitate interpretation of the subsequent results.

Questions:
1. To obtain the specific MTF required for the proposed system, does the optical design depend on the spectral characteristics of the image sensor?
2. The Intensity 3D imaging mode is interesting in that it mitigates imaging speed limitations in DMDs and cameras by employing multiple fast-switching LEDs. In this work, three channels are used to achieve a threefold speed increase. If a snapshot-type spectral camera were used instead, what frame rate could be achieved? What are the potential technical limitations?

3. Related to the above, could future modification of projection optics and reconstruction algorithms enable speed scaling proportional to the number of illumination LEDs beyond three, even when using a conventional RGB camera?
4. The depth resolution error appears to increase gradually with frame rate. What is the dominant cause of this trend? Please clarify whether the cause is common to both color mode and intensity mode, or specific to the intensity mode, where RGB illumination is sequentially applied.
5. The membrane vibration results at ultrasound frequency appear to deviate slightly from an ideal sinusoidal waveform. While this is not problematic, could you provide further quantitative analysis to strengthen the system characterization? Membrane vibration is a reproducible and well-known dynamic target, rather than the static target presented in Supplementary Note 4. In particular, comparing the results (errors) obtained from each of the RGB illumination channels would help evaluate the developed system.

Reviewer #2

(Remarks to the Author)
This manuscript develops a chromatically band-limited illumination profilometry (C-BLIP). It projects programmable grayscale fringes at ultrahigh speed by synergizing a DMD with a user-selectable encoding region of interest and a custom-designed projection lens for chromatically band-limited illumination and acquires spatially multiplexed color fringe images to achieve 3D image reconstruction. It's meaningful and useful in high-speed dynamic 3D measurement. However, some important issues are not clearly addressed. I don't think it suitable for publication in Nature Communications at current version.
1.What's really conventional FPP? Because conventional FPP is the most related research background, it is strongly recommended to give a comprehensive and detail review about conventional FPP at first. With the development of science and technology, conventional FPP has developed many methods, such as Fourier transformation profilometry(FTP), phase-shifting profilometry(PSP), Moiré profilometry(MP), computer-generated Moiré profilometry (CGMP), modulation measuring profilometry(MMP), Phase-differencing profilometry(PDP), empirical mode decomposition(EMD) based profilometry, video projection based profilometry and deep-learning based profilometry(such as fringe projection profilometry by multi-attention fringe restoration network, deep Bayesian inference based profilometry). In this way, 'Akin to conventional FPP' will naturally follow. The more comprehensive the FPP review can be, the more scientific importance and acadmic significance of your manuscript will be.
2. After above comprehensive conventional FPP review, 'phase-shifting FPP ' may be naturally replaced by 'PSP'.
3. Due to arctangent operation in above introduced profilometries, the resolved phase is wrapped within (-π,π], so phase unwrapping is a indispensable and important step. But the authors haven't given a review about phase unwrapping in Introduction. Phase unwrapping includes spatial phase unwrapping(SPU) and temporal phase unwrapping(TPU). At least the quality-guided SPU, the branch-cut SPU, diamond type SPU, curtain type SPU and so on should be introduced and cited in SPU and at least dual-frequency (wavelength) hierarchical TPU, dual-frequency (wavelength) heterodyne TPU, Gray code, fringe amplitude encoding TPU, phase-intensity hybrid encoding TPU, binary coding TPU and phase coding TPU, phase shift coding TPU, phase shift coding division multiplexing TPU, symmetry multi-level Gray coding TPU, order shifting TPU and so on should be introduced and cited in TPU. Then your selection 'branch cut phase unwrapping algorithm 'naturally becomes persuasive.
4. Phase resolving, phase unwrapping and phase-to-height mapping are three key and most important steps in phase based FPP methods. However, only phase resolving is shown in 'METHODS'. It is strongly recommended to establish a complete mathematical model by adding both phase unwrap and phase-to-height mapping in 'METHODS'.
5. I think the chapter order should be 'Introduction, Methods, Experimental Results, Discussion, and Conclusion'. If the current chapter order is required by the magazine, this clause can be ignored. Otherwise, please rearrange the chapter order and add a 'Conclusion'.
6. In 'RESULTS', a static accuracy verification experimental result should be shown. For instance, a standard ball measuring results with MAE and RMSE analysis.
7. Because you select branch-cut SPU to achieve phase unwrapping, it hard to image how you figure out the multiple isolated objects like Fig.2(a) and Fig.2 (b). Please address it more detail.
8. Are there any limitations to the proposed method, and what are the directions for improvement? Please clarify it in the Conclusion or a proper position in the context.

Reviewer #3

(Remarks to the Author)
This paper presents an interesting and well-engineered approach for ultrahigh-speed 3D surface imaging based on chromatically band-limited illumination. By jointly optimizing projection (via DMD ROI encoding and custom optical design) and detection (via spatial–spectral multiplexing on a color camera), the authors significantly push the limits of single-shot fringe projection profilometry (SS-FPP). The demonstrated frame rates (up to 150 kfps) and continuous-streaming capability are particularly impressive.
The manuscript is generally well written, technically sound, and experimentally convincing. The proposed C-BLIP framework shows potential for applications in high-speed metrology, biomechanics, and data security. However, several important technical and conceptual issues require further clarification and discussion before the manuscript can be considered for publication.
Major Comments:
1. One of the key claimed contributions is the multiplexing of fringe information in both spectral and color domains, achieving up to a 12:1 compression ratio within a single exposure. However, in its current form, this strategy is more accurately described as multiplexing rather than true compressive sensing. From my perspective, the system does not yet exploit

sparsity priors or reconstruction algorithms typical of compressive sensing frameworks. It would be valuable to discuss whether the current C-BLIP architecture could be extended to incorporate compressive sensing strategies (e.g., random coding, optimized measurement matrices, or learning-based reconstruction), potentially enabling higher compression ratios and single-shot compressed ultrafast 3D imaging.
2. The manuscript demonstrates frequency multiplexing of multiple fringe patterns, but does not clearly analyze its theoretical or practical upper bound. What fundamentally limits the number of multiplexed frequencies? Is it constrained by the system MTF, spectral bandwidth of LEDs, camera sampling, or noise floor? A quantitative discussion or guideline would significantly strengthen the paper.
3. The projected fringe frequencies appear relatively low. For objects with complex geometry, high curvature, or strong surface texture, spectral overlap and aliasing may become significant. How robust is the proposed spectral separation under such conditions? Are there safeguards to ensure reliable phase retrieval? This issue is critical for real-world applicability and should be addressed.
4. The manuscript indicates that phase is extracted from multiplexed single-shot data and unwrapped independently for each time instance. A key concern is temporal consistency: How is the consistency of phase unwrapping ensured across different time frames? How is the reference phase (or starting point) aligned temporally? Is there any risk of frame-to-frame ambiguity or phase jumps? Without proper temporal constraints, dynamic 3D reconstruction may suffer from discontinuities. The authors are encouraged to clarify this point or provide a stabilization strategy.
5. The ultrasound audio encryption experiment is particularly interesting and demonstrates the versatility of the system. This naturally raises a broader question: Could this technique be extended to passive acoustic sensing or eavesdropping, for example, by recovering audio signals from vibration-induced motion (e.g., curtains or lightweight structures)? Is the sensitivity and bandwidth of C-BLIP sufficient for such applications? While this may be beyond the scope of the current work, a brief discussion would enrich the impact of the paper.

Version 1:

Reviewer comments:

Reviewer #1

(Remarks to the Author)
My comments have been well addressed.

Reviewer #2

(Remarks to the Author)
This manuscript develops a chromatically band-limited illumination profilometry (C-BLIP). It projects programmable grayscale fringes at ultrahigh speed by synergizing a DMD with a user-selectable encoding region of interest and a custom-designed projection lens for chromatically band-limited illumination and acquires spatially multiplexed color fringe images to achieve 3D image reconstruction. It's meaningful and useful in high speed dynamic 3D measurement.The authors have revised this manuscript according to the comment.I think it suitable for publication in Nature Communications if some minor revisions can be done.
1.Because phase unwrapping is very important in all profilometries, While temporal phase unwrapping is first occurs in the main context,it's strongly recommended to cite some latest literature about temporal phase unwrapping especially those with the least additional patterns or even without any additional pattern for further enhancing academic value.
2.Though literature [18] is cited while spacial phase unwrapping is first occurs in the main context,it's not typical at least phase unwrapping isn't in its title. It's strongly recommended to cite some latest literature about spacial phase unwrapping especially those reflect path-dependence feature, such as diamond(rhombus) type SPU, curtain type SPU reflecting path shape, for further enhancing academic value.

Reviewer #3

(Remarks to the Author)
The authors have adequately addressed all of my previous concerns. In particular, the newly added experiment on ultrasound-encoded audio recovery provides an important and convincing validation of the proposed method and substantially strengthens the manuscript.
I am satisfied with the authors' responses and revisions. I therefore have no further concerns and recommend that the manuscript be accepted for publication.

Open Access This Peer Review File is licensed under a Creative Commons Attribution 4.0 International License, which permits use, sharing, adaptation, distribution and reproduction in any medium or format, as long as you give appropriate credit to the original author(s) and the source, provide a link to the Creative Commons license, and indicate if changes were made.
In cases where reviewers are anonymous, credit should be given to 'Anonymous Referee' and the source.
The images or other third party material in this Peer Review File are included in the article's Creative Commons license, unless indicated otherwise in a credit line to the material. If material is not included in the article's Creative Commons license and your intended use is not permitted by statutory regulation or exceeds the permitted use, you will need to obtain permission directly from the copyright holder.
To view a copy of this license, visit https://creativecommons.org/licenses/by/4.0/

Response to Reviewers' Comments

We sincerely appreciate the reviewers for their thorough reviews of our work and their insightful and constructive comments, which have helped greatly improve the quality of our manuscript. In this round of revision, we have included detailed descriptions and illustrations to clarify the technical features of our work and strengthen the corresponding analyses. We have also conducted a new experiment to directly address the reviewers' concerns.

In Main Text, these amendments are reflected in the following major changes:

- We have revised Introduction to expand the review of fringe projection profilometry and its phase extraction methods.
- We have added a new subsection and Fig. 6 in Results to describe the new experiment.
- We have updated Methods to include a complete mathematical model for the three-dimensional (3D) reconstruction of chromatically band-limited illumination profilometry (C-BLIP).

As for Supplementary Materials, the major changes include:

- We have added Supplementary Notes 1 and 2 to provide a comprehensive review of fringe projection profilometry and its phase extraction methods.
- We have reorganized Supplementary Note 4, relocating the description of the phase-to-depth calculation to Methods in Main Text.
- We have added Supplementary Note 8 and Supplementary Movie 8 to describe the details of the new experiment.

In the following, we provide point-by-point responses to the reviewers' comments. The changes in the revised manuscript are highlighted in red.

Reviewer #1

[Comment 0]

The authors presented a novel three-dimensional shape measurement technique named chromatically band-limited illumination profilometry. The method aims to overcome a limitation of conventional 3D profilometry, namely, measurement speed. By combining RGB LED illumination with a high-speed DMD for fringe generation and a carefully designed projection lens system, the authors project fringe patterns onto the object and reconstruct 3D information from camera-captured images. To highlight the capability of the proposed method, the authors presented imaging and analysis demonstrations of fast dynamics such as a turbofan blade-projectile collision and maple-samara fall aerodynamics. While binary patterns generated by DMDs are generally inferior in spatial quality compared to those produced by liquid-crystal SLMs, DMDs offer significantly higher switching speeds. In this work, the authors cleverly introduce an intentional spatial-frequency limitation in the projection optics to improve fringe quality, effectively resolving this trade-off. This design strategy is particularly compelling. In addition, the system description provided in the Supplementary Information is thorough and detailed. It presents a technical platform that is likely to stimulate derivative research and practical extensions. Overall, this study is interesting and significant. This manuscript presents sufficient analysis and experimental validation to substantiate the proposed method. Therefore, I recommend publishing this work in Nature Communications, provided that the following comments are addressed.

[Response 0]

We thank the reviewer for recognizing the novelty and the significance of our work, the merits of our design strategy, and the thorough system description and experimental validation presented in the manuscript.

[Comment 1]

Major comments: The technical contribution of this work is clear and compelling. To further strengthen the manuscript, I suggest clarifying the core innovation more explicitly in the main text. After introducing the system configuration in the Results section, the manuscript proceeds directly to multiple demonstrations. It may be beneficial to more explicitly highlight the principal advantages of the proposed method before presenting these demonstrations, as this would help guide readers on what aspects to focus on when interpreting the subsequent imaging results. As

high-speed operation is emphasized in the Abstract and Introduction, this clarification could particularly include concise quantitative statements regarding the frame rates and related parameters, as well as a clear explanation of how the proposed design overcomes the bottlenecks of conventional methods.

[Response 1]

We agree that explicitly restating the core innovation and principal advantages in Results will help guide readers before presenting the demonstrations. We would like to point out that the principal advantages of C-BLIP, together with how the design addresses the key bottlenecks of conventional methods, are introduced in the last paragraph of Introduction in the original manuscript (now in Lines 73–79 in the revised manuscript).

To further address the reviewer's suggestion, we have now added a paragraph in the subsection "Operating principle" in Results, immediately before the demonstrations (see Lines 116–125 in Main Text), restating how C-BLIP's image-acquisition framework and image-recovery paradigm overcome the key bottlenecks of existing time-resolved single-shot fringe-projection profilometry (SS-FPP) and previewing the subsequent demonstrations together with their frame rates.

[Comment 2]

In addition, a brief summary of the projection lens design principles in the main text could further enhance the readability and impact of the paper.

[Response 2]

We agree that a concise summary of the projection lens design principle in Main Text would improve the readability and impact of the paper. We have briefly expanded the description in the subsection "Operating principle" (see Lines 94–95 in Main Text) to more explicitly summarize the lens design principle. The detailed design process—including the design objectives, modulation transfer function (MTF) optimization, and lens configuration—remains in Methods and Supplementary Note 3.

[Comment 3]

Minor Comment: Regarding system resolution, the Results section refers readers to Supplementary Note 4. It would be helpful to include values under specific conditions directly in this paragraph, as this would facilitate interpretation of the subsequent results.

[Response 3]

We have now reported the depth resolution under each specific operating condition directly within the corresponding subsections in Results. Please see Lines 145, 165, and 204 in Main Text.

[Comment 4]

Questions: 1. To obtain the specific MTF required for the proposed system, does the optical design depend on the spectral characteristics of the image sensor?

[Response 4]

We would like to clarify that the wavelength-dependent MTF is specified entirely on the projection side and is independent of the image sensor's spectral characteristics. The design is mainly determined by the emission spectra of the incoherent light-emitting diodes (LEDs) and the spatial frequencies set by the digital micromirror device (DMD) pixel and pitch sizes. The used F/2.0 camera lens and the image sensor (with a 10-µm pixel size) are capable of high-quality imaging of the projected fringes over the entire spectral band of all three LEDs. Therefore, the spectral response of the image sensor plays no role in obtaining the specific MTF required for the proposed system.

[Comment 5]

2. The Intensity 3D imaging mode is interesting in that it mitigates imaging speed limitations in DMDs and cameras by employing multiple fast-switching LEDs. In this work, three channels are used to achieve a threefold speed increase. If a snapshot-type spectral camera were used instead, what frame rate could be achieved? What are the potential technical limitations?

[Response 5]

We thank the reviewer for this insightful question. The threefold speed increase in the intensity 3D imaging mode arises from three spectrally distinct LED illumination channels. Consequently, replacing the color camera with a snapshot-type spectral camera while retaining the present three-LED scheme would not increase C-BLIP's frame rate.

To exceed the imaging speed, one could increase the number of spectrally distinct illumination sources and use a snapshot spectral camera (e.g., Photonfocus, MV0-D2048x1088-C01-HS03-G2) with matching detection channels or use spectral unmixing as a reconstruction approach (as detailed in our responses to Reviewer 3's Comments 1 and 2). This point has been added to Discussion in the revised manuscript (see Lines 287–289 and Lines 298–299 in Main Text).

In practice, however, this scaling could be bounded by several technical limitations. The LED emission spectrum has a certain bandwidth, which limits the maximum number of resolvable color channels. More color channels also indicate a reduced illumination time of each LED (i.e., t_{L}) for a given DMD's display time (i.e., t_{D}, see Supplementary Fig. 3c). In this regard, the LED's modulation bandwidth would ultimately determine the maximum imaging speed. This operation would also induce undesired byproducts, including spectral crosstalk, reduced signal-to-noise ratio (SNR), reduced spatial resolution by the snapshot spectral camera, and increased system complexity, all of which would reduce the fidelity of reconstructing time-resolved 3D spatial information at high speeds.

[Comment 6]

3. Related to the above, could future modification of projection optics and reconstruction algorithms enable speed scaling proportional to the number of illumination LEDs beyond three, even when using a conventional RGB camera?

[Response 6]

Modification of projection optics could enhance the color projection ability. As we have responded to this reviewer's Comment 5, the implementation of multiple spectrum-separated LEDs with a snapshot spectral camera could increase the number of spectral bands. Provided sufficient modulation bandwidth of these LEDs to support the corresponding t_{L} within t_{D}, C-BLIP's speed can be scaled proportionally to the number of illumination LEDs.

As the reviewer pointed out, the implementation of advanced reconstruction algorithms may enable speed enhancement with a conventional RGB camera. Given the knowledge of LEDs' illumination spectra and the camera's spectral response, the compressively recorded spectra can be recovered by solving a constrained optimization problem with appropriate priors (e.g., non-negativity, spectral smoothness, or sparsity), as demonstrated for LED-illumination multispectral

imaging with RGB sensors [R2]. The achievable number of channels in this case is set by the conditioning of the inverse problem and the available SNR. In practice, a 2× increase would be expected without decreasing the reconstruction fidelity [R3].

We thank the reviewer for pointing to a promising direction for further development. These points have been added to the revised manuscript (see Lines 287–292 in Main Text).

[Comment 7]

4. The depth resolution error appears to increase gradually with frame rate. What is the dominant cause of this trend? Please clarify whether the cause is common to both color mode and intensity mode, or specific to the intensity mode, where RGB illumination is sequentially applied.

[Response 7]

The frame-rate-dependent depth resolution applies to both the color-3D-imaging and the intensity-3D-imaging modes. The dominant cause is the lower SNR of each reconstructed frame at higher imaging speeds. The lower SNR propagates through the Fourier-transform profilometry (FTP) phase extraction into a noisier phase map, which manifests as a larger error from the ground truth (shown in Supplementary Fig. 4). The sequential RGB illumination used in the intensity 3D imaging mode is not the dominant cause because each illumination produces one fringe image, and C-BLIP relies on single frames for 3D reconstruction.

[Comment 8]

5. The membrane vibration results at ultrasound frequency appear to deviate slightly from an ideal sinusoidal waveform. While this is not problematic, could you provide further quantitative analysis to strengthen the system characterization? Membrane vibration is a reproducible and well-known dynamic target, rather than the static target presented in Supplementary Note 4. In particular, comparing the results (errors) obtained from each of the RGB illumination channels would help evaluate the developed system.

[Response 8]

We performed a channel-wise analysis on the datasets in Figs. 5c (M_1, a dual-tone beat signal) and 5d (M_2, a linearly chirped signal) of Main Text. For each speaker, as displayed in Fig. R1a, we separately extracted the reconstructed depth information corresponding to the red, green, and blue illumination channels in the same central region of interest. We then fit the full 3D reconstructed

depth information to the analytical input model, evaluated the fitted reference at each channel's acquisition time points, and computed the per-channel normalized root-mean-square error (NRMSE) of the measurements against the fitted reference (Fig. R1b). Expressed as a percentage of the peak-to-peak depth difference of the fitted reference, the NRMSE stays at a low level across all three color channels. This accuracy is comparable to C-BLIP's depth resolution relative to the measured depth range (Supplementary Note 6). The small inter-channel differences may arise from certain experimental factors. First, the membrane vibration may deviate from the input waveform, which can vary cycle by cycle. Second, the deployed camera has a decreasing spectral responsiveness towards long wavelengths. The varied SNRs may result in slightly different reconstruction quality.

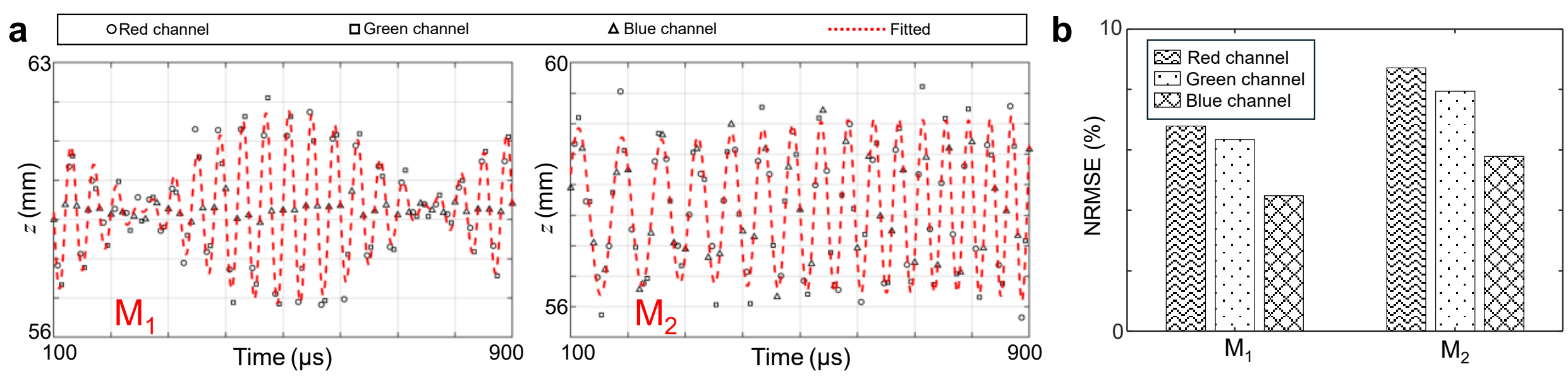

Fig. R1. Analysis of C-BLIP reconstruction across three color channels for dynamic membranes' vibration. a Reconstructed depth from the red, green, and blue illumination channels of M_1 (left) and M_2 (right), overlaid with the fitted analytical input model (red dashed lines). **b** Channel-wise normalized root-mean-square error (NRMSE) of the reconstructed depth against the fitted references.

Reviewer #2

[Comment 0]

This manuscript develops a chromatically band-limited illumination profilometry (C-BLIP). It projects programmable grayscale fringes at ultrahigh speed by synergizing a DMD with a user-selectable encoding region of interest and a custom-designed projection lens for chromatically band-limited illumination and acquires spatially multiplexed color fringe images to achieve 3D image reconstruction. It's meaningful and useful in high-speed dynamic 3D measurement. However, some important issues are not clearly addressed. I don't think it suitable for publication in Nature Communications at current version.

[Response 0]

We thank the reviewer for recognizing C-BLIP as a meaningful and useful approach for high-speed dynamic 3D measurement. To address the reviewer's concerns and comments, we have substantially revised the Main Text and Supplementary Notes, as detailed in the point-by-point responses below.

[Comment 1]

1.What's really conventional FPP? Because conventional FPP is the most related research background, it is strongly recommended to give a comprehensive and detail review about conventional FPP at first. With the development of science and technology, conventional FPP has developed many methods, such as Fourier transformation profilometry(FTP), phase-shifting profilometry(PSP), Moiré profilometry(MP), computer-generated Moiré profilometry (CGMP), modulation measuring profilometry(MMP), Phase-differencing profilometry(PDP), empirical mode decomposition(EMD) based profilometry, video projection based profilometry and deep-learning based profilometry(such as fringe projection profilometry by multi-attention fringe restoration network, deep Bayesian inference based profilometry). In this way, 'Akin to conventional FPP' will naturally follow. The more comprehensive the FPP review can be, the more scientific importance and academic significance of your manuscript will be.

[Response 1]

We agree that a clearer review of conventional FPP would help readers better understand the technical foundation and significance of C-BLIP. Following the reviewer's suggestion, we have expanded the first paragraph of the Introduction (Lines 27–43) into a structured overview of

conventional FPP, organized by how the fringes are generated and how their phase is extracted. Meanwhile, we have added Supplementary Note 1, which provides additional details of each conventional FPP method mentioned above. In restructuring this review, we particularly benefited from the comprehensive overview of FPP by Zhang L., Ji Y., Wu W., and Wang J. (Precision Engineering 96, 80-93, 2025), which we have cited as Ref. 15 in the revised Main Text. Other newly cited references in Main Text and Supplementary Materials include:

- Fourier transformation profilometry
  - Takeda, M. and Mutoh, K. Fourier transform profilometry for the automatic measurement of 3-D object shapes. Applied Optics 22, 3977-3982 (1983).
  - Su, X. and Chen, W. Fourier transform profilometry: a review. Optics and Lasers in Engineering 35, 263-284 (2001).
- Phase-shifting profilometry
  - Feng, S., Zuo, C., Tao, T., Hu, Y., Zhang, M., Chen, Q. and Gu, G., Robust dynamic 3-D measurements with motion-compensated phase-shifting profilometry. Optics and Lasers in Engineering 103, 127-138 (2018).
  - Xu, P., Liu, J. and Wang, J. High dynamic range 3D measurement technique based on adaptive fringe projection and curve fitting. Applied Optics 62, 3265-3274 (2023).
  - Wang, J., Yang, Y., Xu, P. and Liu, J. Saturation-induced phase error correction method in 3-D measurement based on inverted fringes. Applied Optics 62, 492-499 (2023).
  - Wang, J. and Yang, Y. A new method for high dynamic range 3D measurement combining adaptive fringe projection and original-inverse fringe projection. Optics and Lasers in Engineering 163, 107490 (2023).
- Moiré profilometry:
  - Takasaki, H. Moiré topography. Applied Optics 9, 1467-1472 (1970).
- Computer-generated moiré profilometry:
  - Li, C., Cao, Y., Wang, L., Wan, Y., Fu, G., Wang, Y. and Chen, C., High precision computer-generated moiré profilometry. Scientific Reports 9, 7804 (2019).
- Modulation measuring profilometry:
  - Su, L., Su, X., Li, W. and Xiang, L. Application of modulation measurement profilometry to objects with surface holes. Applied Optics 38, 1153-1158 (1999).
- Phase-differencing profilometry:
  - Wei, Z., Cao, Y., Wu, H., Xu, C., Ruan, G., Wu, F. and Li, C., Dynamic phase-differencing profilometry with number-theoretical phase unwrapping and interleaved projection. Optics Express 32, 19578-19593 (2024).
- Empirical mode decomposition:
  - Zheng, S. and Cao, Y. Fringe-projection profilometry based on two-dimensional empirical mode decomposition. Applied Optics 52, 7648-7653 (2013).
- Video projection based profilometry:
  - Zhang, Y., Cao, Y., Wu, H., An, H. and Huang, X. Real-time profilometry by bicolor grating video projection. Plos One 16, e0259569 (2021).

- Deep learning based profilometry:
  - Zuo, C., Qian, J., Feng, S., Yin, W., Li, Y., Fan, P., Han, J., Qian, K. and Chen, Q., Deep learning in optical metrology: a review. Light: Science & Applications 11, 39 (2022).
  - Qin, J., Jiang, Y., Cao, Y. and Wu, H. Single-shot phase-shifting composition fringe projection profilometry by multi-attention fringe restoration network. Neurocomputing 634, 129908 (2025).
  - Ji, Y., Zhang, L., Wu, W. and Wang, J. Deep learning-based single-frame depth reconstruction empowered by indirect mapping. IEEE Transactions on Instrumentation and Measurement 74, 7010711 (2025).
  - Wang, J., Zhang, S. and Yang, Y. Deep learning-based high dynamic range 3D measurement: The combination of GAN and U-Net. Sensors and Actuators A: Physical 399, 117405 (2026).
  - Tan, J., Niu, H., Su, W. and He, Z. Structured light 3D shape measurement for translucent media base on deep Bayesian inference. Optics & Laser Technology 181, 111758 (2025).
  - Chen, W., Feng, S., Yin, W., Li, Y., Qian, J., Chen, Q. and Zuo, C., Deep-learning-enabled temporally super-resolved multiplexed fringe projection profilometry: high-speed kHz 3D imaging with low-speed camera. PhotoniX 5, 25 (2024).

[Comment 2]

2. After above comprehensive conventional FPP review, 'phase-shifting FPP ' may be naturally replaced by 'PSP'.

[Response 2]

In the revised Introduction (Line 36–37), "phase-shifting FPP" has been replaced by "phase-shifting profilometry". Since this term appears only once in Main Text, we have retained it in full rather than introducing an acronym.

[Comment 3]

3. Due to arctangent operation in above introduced profilometries, the resolved phase is wrapped within (-π,π], so phase unwrapping is a indispensable and important step. But the authors haven't given a review about phase unwrapping in Introduction. Phase unwrapping includes spatial phase unwrapping(SPU) and temporal phase unwrapping(TPU). At least the quality-guided SPU, the branch-cut SPU, diamond type SPU, curtain type SPU and so on should be introduced and cited in SPU and at least dual-frequency (wavelength) hierarchical TPU, dual-frequency (wavelength) heterodyne TPU, Gray code, fringe amplitude encoding TPU, phase-intensity hybrid encoding TPU, binary coding TPU and phase coding TPU, phase shift coding TPU, phase shift coding division multiplexing TPU, symmetry multi-level Gray coding TPU, order shifting TPU and so on

should be introduced and cited in TPU. Then your selection ‘branch cut phase unwrapping algorithm ‘naturally becomes persuasive.

[Response 3]

We agree that phase unwrapping is an indispensable step and that a clear review strengthens the manuscript. Following the reviewer’s suggestion, in the revised Introduction (Lines 35–41), we have reviewed phase unwrapping based on the categorization of temporal phase unwrapping (TPU) and spatial phase unwrapping (SPU). Nonetheless, to avoid overloading Introduction, we added Supplementary Note 2 to cover more details of both categories and all the methods noted by the reviewer.

The newly cited references in Main Text and/or Supplementary Materials include:

- TPU
    - Li, E., Peng, X., Xi, J., Chicharo, J.F., Yao, J. and Zhang, D., Multi-frequency and multiple phase-shift sinusoidal fringe projection for 3D profilometry. Optics Express 13, 1561-1569 (2005).
    - Zuo, C., Huang, L., Zhang, M., Chen, Q. and Asundi, A. Temporal phase unwrapping algorithms for fringe projection profilometry: A comparative review. Optics and Lasers in Engineering 85, 84-103 (2016).
    - Song, L., Zhang, X., Huang, H., Guo, Q., Yang, Y. & Zhu, X. Phase unwrapping method combining dual-frequency heterodyne with dual complementary phase encoding. Optical Engineering 60, 085106 (2021).
    - He, X. and Kemao, Q. A comparison of n-ary simple code and n-ary gray code phase unwrapping in high-speed fringe projection profilometry. Optics and Lasers in Engineering 128, 106046 (2020).
    - Wu, H., Cao, Y., Dai, Y. and Zhang, H. Ultra-fast 3D imaging by a big codewords space division multiplexing binary coding. Optics Letters 48, 2793-2796 (2023).
    - Wang, Y. and Zhang, S. Novel phase-coding method for absolute phase retrieval. Optics Letters 37, 2067-2069 (2012).
    - An, H., Cao, Y., Li, H. and Zhang, H. High-speed 3-D reconstruction based on phase shift coding and interleaved projection. Expert Systems with Applications 234, 121067 (2023).
    - Wu, F., Cao, Y., An, H. and Wei, Z. Dynamic three-dimensional reconstruction with phase shift coding division multiplexing. Sensors and Actuators A: Physical 379, 115847 (2024).
    - Wang, J., Cao, Y., Wu, H. and Wei, Z. Absolute phase retrieval based on fringe amplitude encoding without any additional auxiliary pattern. Optics Express 31, 41952-41966 (2023).
    - Luo, J., Cao, Y. and Wang, J. Absolute phase retrieval based on phase-intensity hybrid encoding without any additional pattern. Optics and Lasers in Engineering 190, 108968 (2025).
    - Li, Y., Cao, Y. and Ran, L. Spatial symmetric multi-level coding absolute phase retrieval for color 3D real-time measurement. Measurement 257, 119017 (2025).
    - Qin, J., Jiang, Y. and Cao, Y. Order-shifting temporal phase unwrapping using uneven duty gray-code for high-accurate and high-efficient 3D measurement. Optics Letters 51, 1472-1475 (2026).

- SPU
  - Wang, J., Yang, Y. and Zhou, Y. Dynamic three-dimensional surface reconstruction approach for continuously deformed objects. IEEE Photonics Journal 13, 1-15 (2021).
  - Su, X. and Chen, W. Reliability-guided phase unwrapping algorithm: a review. Optics and Lasers in Engineering 42, 245-261 (2004).
  - Jiang, H., Xu, Y., Zhang, C., Xu, Z., Huang, J., Tan, H. and Lu, J. An algorithm combining the branch-cut method and rhombus phase unwrapping algorithm. Journal of Physics: Conference Series 1634, 012068 (2020).
  - Xu, C., Cao, Y., Wu, H., Li, H., Zhang, H. and An, H., Curtain-type phase unwrapping algorithm. Optical Engineering 61, 044103 (2022).
  - De Souza, J., Oliveira, M. and Dos Santos, P. Branch-cut algorithm for optical phase unwrapping. Optics Letters 40, 3456-3459 (2015).
  - Goldstein, R. M., Zebker, H. A. and Werner, C. L. Satellite radar interferometry: Two-dimensional phase unwrapping. Radio Science 23, 713-720 (1988).

[Comment 4]

4. Phase resolving, phase unwrapping and phase-to-height mapping are three key and most important steps in phase based FPP methods. However, only phase resolving is shown in 'METHODS'. It is strongly recommended to establish a complete mathematical model by adding both phase unwrap and phase-to-height mapping in 'METHODS'.

[Response 4]

A detailed mathematical model for the phase-to-depth mapping that solves the 3D information was already provided in Supplementary Note 2 in the original Supplementary Materials. Following the reviewer's suggestion, we have moved this part to Methods, where it now joins the phase-resolving and phase-unwrapping steps in the subsection "Image reconstruction of C-BLIP" to provide a complete mathematical description (see Lines 388–404 in Main Text).

[Comment 5]

5. I think the chapter order should be 'Introduction, Methods, Experimental Results, Discussion, and Conclusion'. If the current chapter order is required by the magazine, this clause can be ignored. Otherwise, please rearrange the chapter order and add a 'Conclusion'.

[Response 5]

The current section order follows the Nature Communications author guidelines (https://www.nature.com/ncomms/submit/article), which specify that: "The main text should begin with a section headed Introduction, followed by sections headed Results, Discussion (if appropriate) and Methods (if appropriate)". Thus, we retain the current order.

[Comment 6]

6. In 'RESULTS', a static accuracy verification experimental result should be shown. For instance, a standard ball measuring results with MAE and RMSE analysis.

[Response 6]

It is worth noting that a static accuracy verification is already included in Supplementary Note 6 and Supplementary Fig. 4. For each operating mode, we gave a quantitative assessment and reported the RMSE. Following the reviewer's suggestion, we have also computed and reported the mean absolute error (MAE) in Supplementary Note 6.

Moreover, we would like to point out that the flat reference plane is a standard and widely adopted target for static accuracy verification implemented in many references (e.g., [R4-R8]). We therefore believe that the existing flat-plane verification reported in Supplementary Note 6, with quantitative assessment using both MAE and RMSE, provides the static accuracy assessment requested by the reviewer.

[Comment 7]

7. Because you select branch-cut SPU to achieve phase unwrapping, it hard to image how you figure out the multiple isolated objects like Fig.2(a) and Fig.2 (b). Please address it more detail.

[Response 7]

We agree with the reviewer that the branch-cut algorithm relies on surface continuity and is best suited to connected scenes. We would first point out that the object used to produce Fig. 2a and Fig. 2b is a single continuous half-cube array, whose facets are all physically connected (Fig. R2a) without any isolation or discontinuity. The wrapped phase (shown in Fig. R2b) forms a connected fringe field across the entire array, and the unwrapped phase (shown in Fig. R2c) forms a continuous phase distribution across the structure, confirming that it constitutes one connected domain for branch-cut unwrapping.

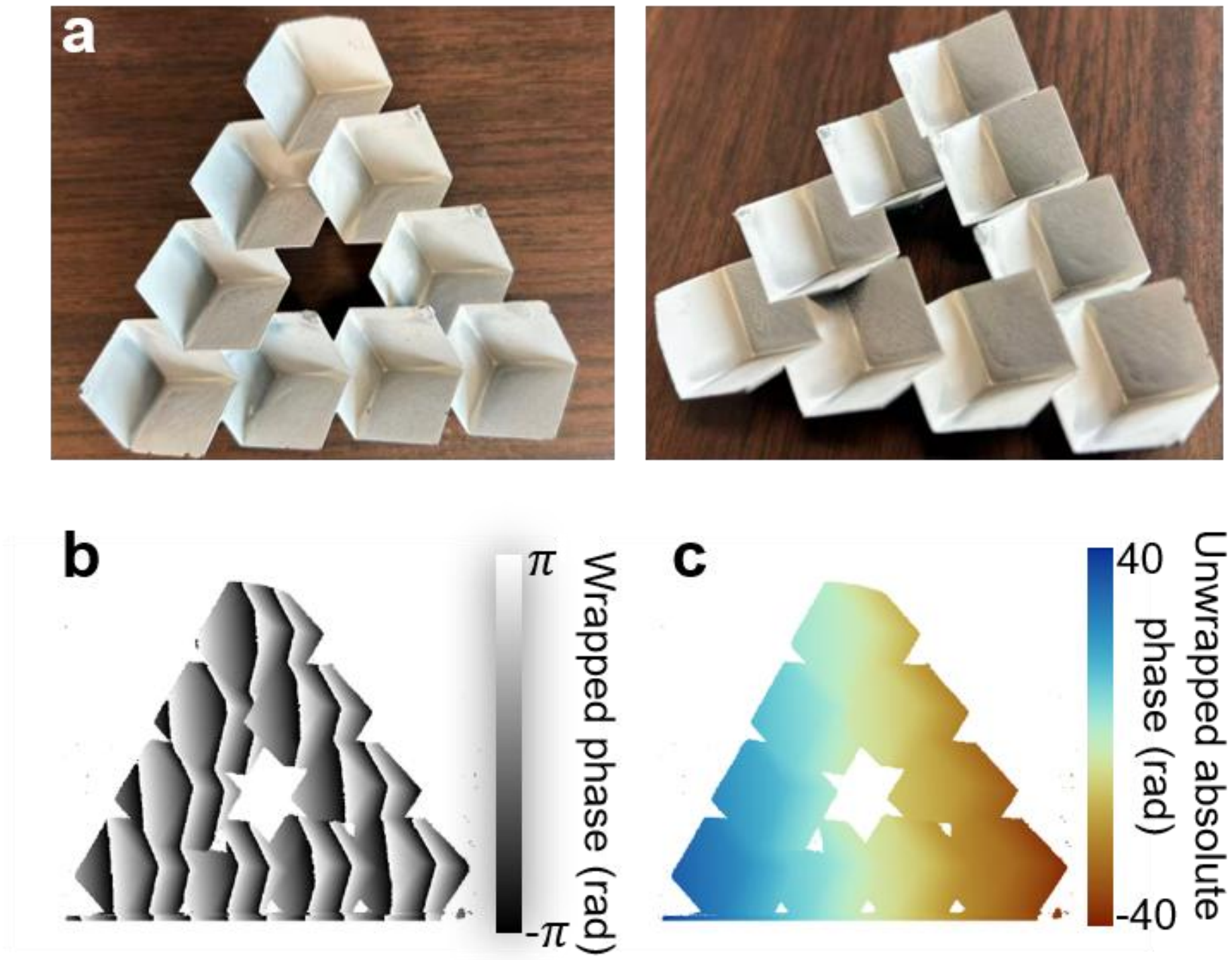

Fig. R2. Surface continuity of the half-cube array used for Fig. 2a–b in Main Text. a 2D photographs. **b-c** Wrapped phase map (b) and unwrapped absolute phase map (c).

[Comment 8]

8. Are there any limitations to the proposed method, and what are the directions for improvement? Please clarify it in the Conclusion or a proper position in the context.

[Response 8]

The current limitations of C-BLIP and the corresponding directions for improvement are included in Discussion in the original manuscript. We have expanded this part in this revision. Please see Lines 287–305 in the revised manuscript.

Reviewer #3

[Comment 0]

This paper presents an interesting and well-engineered approach for ultrahigh-speed 3D surface imaging based on chromatically band-limited illumination. By jointly optimizing projection (via DMD ROI encoding and custom optical design) and detection (via spatial–spectral multiplexing on a color camera), the authors significantly push the limits of single-shot fringe projection profilometry (SS-FPP). The demonstrated frame rates (up to 150 kfps) and continuous-streaming capability are particularly impressive. The manuscript is generally well written, technically sound, and experimentally convincing. The proposed C-BLIP framework shows potential for applications in high-speed metrology, biomechanics, and data security. However, several important technical and conceptual issues require further clarification and discussion before the manuscript can be considered for publication.

[Response 0]

We thank the reviewer for recognizing C-BLIP as an interesting and well-engineered approach that significantly pushes the limits of SS-FPP, and for the positive assessment of our manuscript as well written, technically sound, and experimentally convincing.

[Comment 1]

Major Comments:

1. One of the key claimed contributions is the multiplexing of fringe information in both spectral and color domains, achieving up to a 12:1 compression ratio within a single exposure. However, in its current form, this strategy is more accurately described as multiplexing rather than true compressive sensing. From my perspective, the system does not yet exploit sparsity priors or reconstruction algorithms typical of compressive sensing frameworks. It would be valuable to discuss whether the current C-BLIP architecture could be extended to incorporate compressive sensing strategies (e.g., random coding, optimized measurement matrices, or learning-based reconstruction), potentially enabling higher compression ratios and single-shot compressed ultrafast 3D imaging.

[Response 1]

We agree with the reviewer's characterization that C-BLIP works by spatial frequency multiplexing for each color channel. We do not intend to claim compressive sensing (CS) for C-BLIP.

The current C-BLIP architecture could be extended to incorporate CS strategies. From a hardware point of view, as detailed in our response to Reviewer 1's Comment 6, a potential approach is to use a conventional RGB camera to compressively capture complex illumination from the deployed LEDs, followed by learning-based spectral unmixing to reconstruct more color frames for each fringe image. Based on the literature [R2-R3], a 2× increase would be expected without decreasing the reconstruction fidelity. From a software perspective, a potential approach is to use a video-frame interpolation network (e.g., optical flow [R9], transformer [R10], and diffusion models [R11]) pre-trained on large collections of videos with motion and appearance similar to the dynamics under investigation. With this prior information, these networks can generate intermediate frames by learning the motion between consecutive images. In practice, a 2–4× increase in the number of frames is generally considered reliable. Overall, these two approaches would increase the imaging speed by 4–8× without changing the C-BLIP's architecture. We have added these points to Discussion. Please see Lines 287–291 in the revised Main Text.

[Comment 2]

2. The manuscript demonstrates frequency multiplexing of multiple fringe patterns, but does not clearly analyze its theoretical or practical upper bound. What fundamentally limits the number of multiplexed frequencies? Is it constrained by the system MTF, spectral bandwidth of LEDs, camera sampling, or noise floor? A quantitative discussion or guideline would significantly strengthen the paper.

[Response 2]

In general, the multiplexing upper bound is determined by two factors: (1) the number of usable color channels and (2) the number of separable spatial-frequency carriers within each channel.

The number of color channels would primarily be limited by the LEDs' spectral bandwidth and the camera's spectral resolving power. As detailed in our responses to Reviewer 1's Comment 6, a multi-spectral camera could support a larger number of LEDs with different colors, which could enhance the recorded number of frames within the display time of each fringe pattern and hence increase the imaging speed. Moreover, as stated in our response to this reviewer's Comment 1, CS with spectral unmixing presents another approach without hardware modification. For this work, three LEDs were used to pair with the deployed RGB camera. The current upper bound of color channels is three.

For each color channel, the maximum number of multiplexed fringe frequencies is fundamentally limited by the usable Fourier-domain bandwidth. As the reviewer has pointed out, this bandwidth is jointly determined by the projection MTF, camera sampling, spatial frequency content of the 3D object, carrier-orientation constraints required for triangulation, and the SNR after demultiplexing. In practice, the contrast of the projected sinusoidal fringes directly affects the SNR of the recorded patterns and, consequently, the accuracy of phase recovery and 3D reconstruction. In the following, we provide an example using the most strict imaging condition used in this work.

We adopt an MTF⩾0.8 criterion as a conservative contrast threshold, corresponding to ~18 cycles/mm for all three color channels (see Fig. 1e in Main Text). We have verified that the deployed camera for the C-BLIP system (with a 10-μm pixel pitch and a F/2.0 camera lens) can well resolve this spatial frequency in both imaging modes. Therefore, 18 cycles/mm on the DMD sets the boundary of the usable Fourier space (see Fig. R3). Moreover, we further cap the carrier orientation to $-80° < \theta < 80°$, which avoids near-horizontal fringes that are unsuitable for triangulation-based 3D reconstruction.

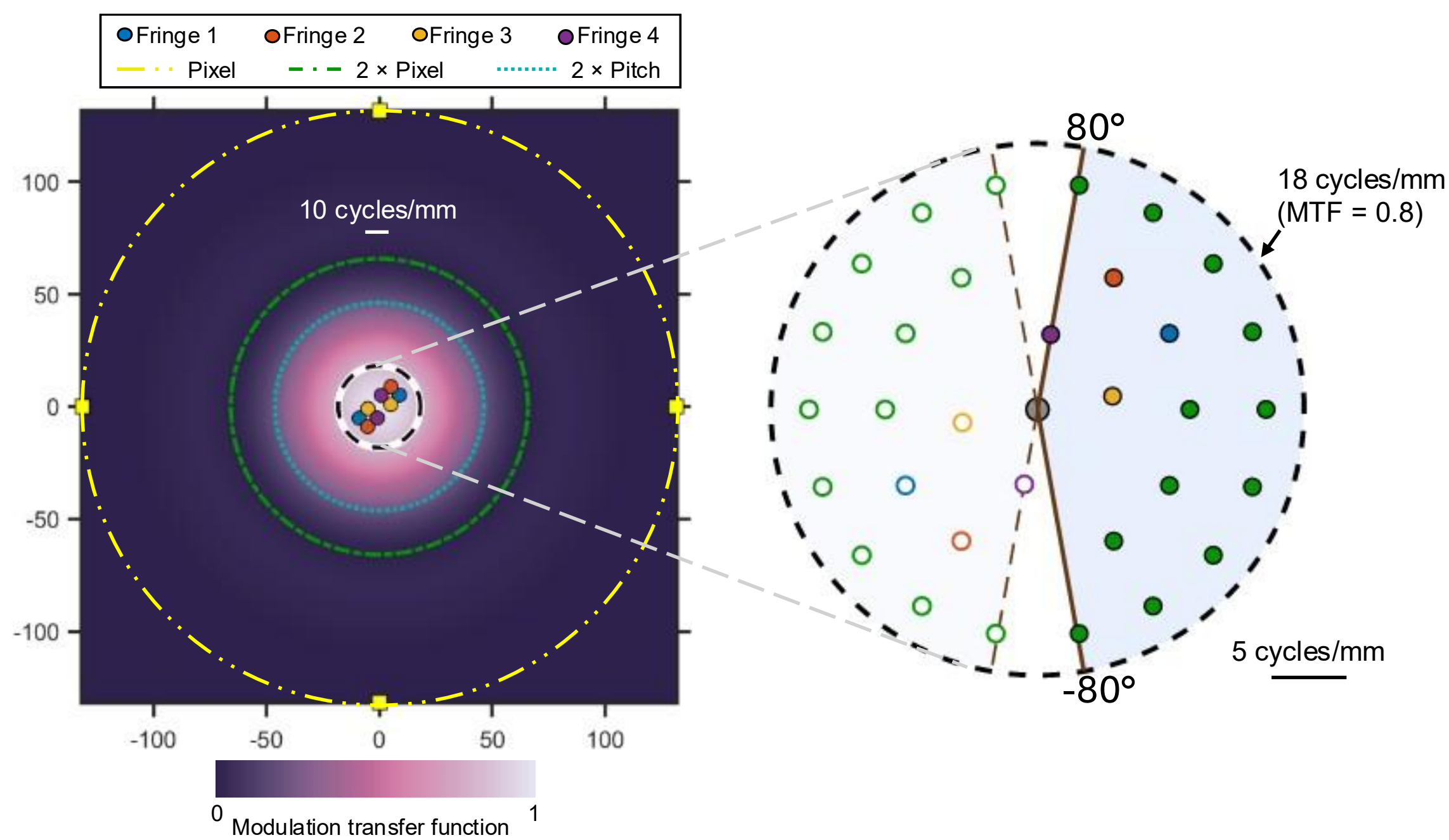

Fig. R3. Illustration of Fourier-domain carrier-packing constraints for estimating the multiplexing capacity. The gray disc represents the dc component. Filled discs represent carrier centers, while open markers represent their conjugate components. Green discs indicate additional candidate carrier centers.

Considering these constraints, the maximum number of separable carrier-frequency channels can be estimated taking into account the four fringe carriers implemented in C-BLIP. From the description in the subsection of "Operating principle" in Results, these carrier frequencies are located at Fringe 1 (ρ_2, θ_1), Fringe 2 (ρ_2,θ_2), Fringe 3 (ρ_1,θ_3), and Fringe 4 (ρ_1,θ_4) where $\rho_1 = 5.14$ cycles/mm, $\rho_2 = 2\rho_1 = 10.29$ cycles/mm, $\theta_1 = 30°$, $\theta_2 = 60°$, $\theta_3 = 10°$, and $\theta_4 = 80°$. By using the same increment between ρ_1 and ρ_2, the radial location of the outermost lobes is determined by $\rho_3 = 3\rho_1 = 15.42$ cycles/mm. Then, the number of lobes is calculated for each radius. In particular, the distance between Fringe 1 and Fringe 2 (i.e., $d_{12} = 5.33$ cycles/mm), which is the shortest amongst any two fringes and more than twice the required Fourier bandwidth of the scene imaged in this study, is used to determine the minimum angular separation by $\Delta\theta_n = 2\sin^{-1}\left[\frac{d_{12}}{2\rho_n}\right]$ $(n = 1, 2, 3)$. At ρ_1, $\Delta\theta_1 = 62.4°$. Considering the center-symmetric nature of the frequency representation, no additional lobe can be added. Following the same calculation, at ρ_2, three lobes can be added to the angles of 0°, -30°, and -60°. At ρ_3, nine lobes can be added to the angles of -79.6°, -59.7°, -39.8°, -19.9°, 0°, 19.9°, 39.8°, 59.7°, and 79.6°. Thus, under the current configuration, a total of 16 carrier channels can be multiplexed per color channel.

It is worth noting that the number of separable carrier channels is scene-dependent. Knowledge of the 3D object's Fourier content serves as an important prior information to guide the projection pattern design for multiplexing without spectral overlapping between adjacent lobes.

We would also like to point out that the exploitation of full spatial frequency multiplexing capacity could bring certain engineering challenges to the current C-BLIP architecture. First, since C-BLIP's imaging speed is jointly determined by the DMD's refresh rate and the LED's illumination time, additional spatial frequency multiplexing would not further enhance the imaging speed. Second, a main motivation of spatial frequency multiplexing is to bypass the limitation in the camera's read-out speed and/or to increase its field of view (FOV). However, the deployed camera in the C-BLIP system has a sufficiently high frame rate to support continuous-streaming operation. At the targeted imaging speeds of this work (i.e., 25–150 kfps), the FOV is limited by the DMD. Thus, additional multiplexing would not enhance the FOV. Third, given the fixed full-well capacity of the deployed camera, increasing the multiplexing number would reduce the effective dynamic range for each fringe pattern and the illumination power. Both actions would result in a lower SNR, which would require a decrease in imaging speed to maintain the measurement accuracy. Considering these reasons, the current operation principle of C-BLIP

distributes the data acquisition workload to both the color spectrum and the spatial frequency. It leverages the color-sensing ability of the RGB camera while retaining the highest possible dynamic range for each multiplexed spatial frequency at the targeted imaging speed. Thus, we believe that the current architecture represents a balanced engineering solution with optimized specifications.

That said, we recognize that higher spatial frequency multiplexing could contribute to the enhancement of imaging capability with some hardware modification. This point is discussed as a future research direction. Please see Lines 295–299 in Main Text.

[Comment 3]

3. The projected fringe frequencies appear relatively low. For objects with complex geometry, high curvature, or strong surface texture, spectral overlap and aliasing may become significant. How robust is the proposed spectral separation under such conditions? Are there safeguards to ensure reliable phase retrieval? This issue is critical for real-world applicability and should be addressed.

[Response 3]

The relatively low projected fringe frequencies were chosen with deliberation. These carriers lie in the higher-MTF-value region of the projection system, which provides better fringe contrast and higher demodulation SNR. For objects with high curvature or steep surface variation, lower fringe frequencies also decrease the probability of phase discontinuities and phase-unwrapping ambiguity.

Objects with complex geometry or strong surface texture can affect the occupied bandwidth in the spatial frequency domain. To ensure robust spectral separation, the safeguards rely on prior knowledge of the measured scene spectrum, which determines how broadly the dc lobe spreads and guides the selection of carrier frequencies. Hence, DMD's programmability for fringe generation serves as another safeguard for C-BLIP. Even for unknown objects, a Fourier analysis could identify spectral overlap and aliasing. This feedback can guide an optimization process to ensure compatibility between the imaging content and the selected fringes. The experimental results shown in our manuscript demonstrate that the current approach of fringe selection can produce high-quality phase reconstruction and, hence, accurate depth measurement.

[Comment 4]

4. The manuscript indicates that phase is extracted from multiplexed single-shot data and unwrapped independently for each time instance. A key concern is temporal consistency: How is

the consistency of phase unwrapping ensured across different time frames? How is the reference phase (or starting point) aligned temporally? Is there any risk of frame-to-frame ambiguity or phase jumps? Without proper temporal constraints, dynamic 3D reconstruction may suffer from discontinuities. The authors are encouraged to clarify this point or provide a stabilization strategy.

[Response 4]

C-BLIP references the unwrapped phase to a preprojected centerline of zero absolute phase for each fringe orientation [Eqs. (6)–(7) in Main Text]. This reference is acquired before the dynamic event and is fixed for every frame. As an SS-FPP method, C-BLIP does not accumulate error over time, which distinguishes it from the multi-shot FPP approaches. Therefore, the phase-unwrapping procedure itself does not introduce cumulative frame-to-frame ambiguity or phase jumps.

In practice, the frame-to-frame inconsistency could be caused by slowly deteriorating system alignment over time. Here, we show that C-BLIP is temporally stable in the time window used for this work. In particular, we measured the depth of a flat surface every 10 seconds over 10 minutes, using the settings for the 150-kfps intensity-only 3D imaging mode, which is the strictest for all results shown in this work. As shown in Fig. R5, the mean depth profile has a standard deviation of 0.02 mm, confirming stable depth reconstruction. As for larger timescales (e.g., months), a routine examination of system alignment and calibration can serve as a common engineering strategy to ensure long-term stability.

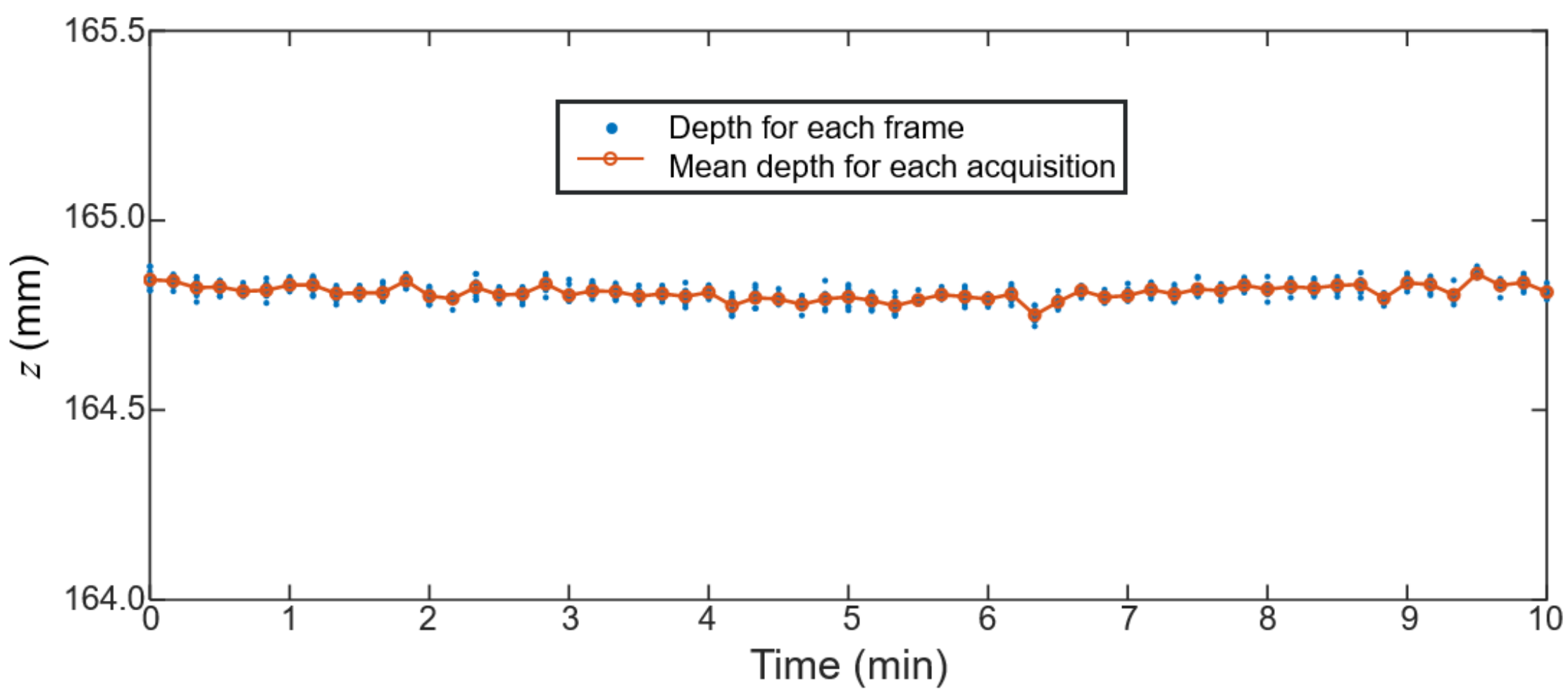

Fig. R5. C-BLIP's stability over a 10-minute measurement window.

[Comment 5]

5. The ultrasound audio encryption experiment is particularly interesting and demonstrates the versatility of the system. This naturally raises a broader question: Could this technique be extended to passive acoustic sensing or eavesdropping, for example, by recovering audio signals from vibration-induced motion (e.g., curtains or lightweight structures)? Is the sensitivity and bandwidth of C-BLIP sufficient for such applications? While this may be beyond the scope of the current work, a brief discussion would enrich the impact of the paper.

[Response 5]

We thank the reviewer for acknowledging that our ultrasound audio encryption experiment is interesting and for the insightful suggestion to further explore the application potential of C-BLIP. In the revised manuscript, we added a new experiment to demonstrate that C-BLIP is capable of recovering audio signals from vibration-induced motion on a curtain-mimicking structure. These results are presented in a new subsection in Results titled "Intensity 3D imaging of ultrasound-encoded audio recovery at 150 kfps" (Lines 240–264), Fig. 6, Supplementary Note 8, and Supplementary Movie 8. We have also added a brief discussion of this capability and its broader implications in the Discussion (Lines 312–314).

References

[R1] Hagen N. and Kudenov M. W., Review of snapshot spectral imaging technologies, Optics Engineering 52, 090901 (2013).

[R2] He T., Zhang Q., Zhou M., Kou T., and Shen J., Single-shot hyperspectral imaging based on dual attention neural network with multi-modal learning, Optics Express 30, 9790-9813 (2022)

[R3] Liang J. Punching holes in light: recent progress in single-shot coded-aperture optical imaging. Reports on Progress in Physics 83, 116101 (2020).

[R4] Rao, G., Song, L., Zhang, S., Yang, X., Chen, K. and Xu, J. Depth-driven variable-frequency sinusoidal fringe pattern for accuracy improvement in fringe projection profilometry. Optics Express 26, 19986-20008 (2018)

[R5] Dashpute, A., Wang, J., Taylor, J. *et al.* Accurate and fast event-based shape measurement of mixed reflectance scenes. Nature Communications 17, 4407 (2026).

[R6] Sun J. and Zhang Q., A 3D shape measurement method for high-reflective surface based on accurate adaptive fringe projection. Optics and Lasers in Engineering 153, 106994 (2022).

[R7] Song H., Kong L., Tang X., Xu X. and Wang B., Extrinsic parameters optimization for fringe projection system based on standard components. Measurement 242, 115836 (2025).

[R8] Peng J., Ouyang H., Yu Q., Yu Y. and Wang K., Phase error correction for fringe projection profilometry by using constrained cubic spline. Advances in Manufacturing 2, 39-47 (2014).

[R9] Jiang, H., Sun, D., Jampani, V., Yang, M.H., Learned-Miller, E. and Kautz, J., Super slomo: High quality estimation of multiple intermediate frames for video interpolation. Proceedings of the IEEE Conference on Computer Vision and Pattern Recognition, 9000-9008 (2018).

[R10] Lu, L., Wu, R., Lin, H., Lu, J. and Jia, J., Video frame interpolation with transformer. Proceedings of the IEEE Conference on Computer Vision and Pattern Recognition, 3532-3542 (2022).

[R11] Jain, S., Watson, D., Tabellion, E., Poole, B. and Kontkanen, J., Video interpolation with diffusion models. Proceedings of the IEEE Conference on Computer Vision and Pattern Recognition, 7341-7351 (2024).

Response to Reviewers' Comments

Reviewer #1

[Comment 0]

My comments have been well addressed.

[Response 0]

We thank the reviewer for acknowledging that all the comments have been well addressed.

Reviewer #2

[Comment 0]

This manuscript develops a chromatically band-limited illumination profilometry (C-BLIP). It projects programmable grayscale fringes at ultrahigh speed by synergizing a DMD with a user-selectable encoding region of interest and a custom-designed projection lens for chromatically band-limited illumination and acquires spatially multiplexed color fringe images to achieve 3D image reconstruction. It's meaningful and useful in high speed dynamic 3D measurement.The authors have revised this manuscript according to the comment.I think it suitable for publication in Nature Communications if some minor revisions can be done.

[Response 0]

We thank the reviewer for recognizing the meaningfulness and usefulness of C-BLIP. We also appreciate the reviewer's positive assessment of our revisions and the manuscript's suitability for publication. We have addressed the reviewer's comments below and made the corresponding revisions to further improve the manuscript.

[Comment 1]

1. Because phase unwrapping is very important in all profilometries, While temporal phase unwrapping is first occurs in the main context,it's strongly recommended to cite some latest literature about temporal phase unwrapping especially those with the least additional patterns or even without any additional pattern for further enhancing academic value.

[Response 1]

We agree that citing the latest developments in temporal phase unwrapping (TPU), particularly those using fewer phase-shifting steps or no auxiliary patterns for fringe-order determination, would further strengthen the review of phase unwrapping in fringe projection profilometry.

In the previous revision, we had already highlighted TPU developments that reduce the number of auxiliary patterns and cited representative literature in Supplementary Note 2 (Lines 77–82). Following the reviewer's suggestion, we have added two recent and representative studies that directly address the two cases raised by the reviewer:

(1) Wang et al. (2025) reduce the number of phase-shifting patterns required for multi-frequency TPU [*Wang, J., Ji, Y., Zhang, L. & Wu, W. Fringe projection 3D imaging optimized for accuracy and efficiency. Optics Express 33, 54709–54732 (2025)*];

(2) Yuan et al. (2026) demonstrate single-frequency TPU without auxiliary patterns for fringe-order determination [*Yuan, H., Wang, J., Xing, H., Zeng, H., Wei, C., Peng, R., Yue, H. & Liu, Y. Single-frequency temporal phase unwrapping without additional patterns for 3D shape measurement. Optics & Laser Technology 204, 115951 (2026)*].

These references have been added at the first occurrence of temporal phase unwrapping in the Main Text (Line 38, as References 14 and 15), and the corresponding discussion has been added to Supplementary Note 2 (Lines 72–76).

[Comment 2]

2. Though literature [18] is cited while spacial phase unwrapping is first occurs in the main context,it's not typical at least phase unwrapping isn't in its title. It's strongly recommended to cite some latest literature about spacial phase unwrapping especially those reflect path-dependence feature, such as diamond(rhombus) type SPU, curtain type SPU reflecting path shape, for further enhancing academic value.

[Response 2]

We agree that citing more representative and recent literature on spatial phase unwrapping (SPU), particularly studies reflecting its path-dependent characteristics, would further enhance the academic value of the manuscript.

In the previous revision, we had emphasized that SPU methods differ mainly in how the unwrapping path is selected to prevent error propagation, and specifically discussed diamond/rhombus-type (Line 90 in Supplementary Note 2 with Supplementary Reference 40) and

curtain-type path geometries (Line 91 in Supplementary Note 2 with Supplementary Reference 41). Following the reviewer's suggestion, we have now cited these representative studies at the first occurrence of spatial phase unwrapping in the Main Text (Line 41, as References 21 and 22). In addition, we have added one recent SPU study [*Zhang, Y., Cao, Y., Wu, H. & Li, C. Spatial phase unwrapping algorithm based on multi-anchors bidirectional location and suppression. Optical Engineering 63, 014101 (2024)*], as Reference 23, which further illustrates the path-dependent nature of SPU through a multi-anchor bidirectional unwrapping strategy, with the corresponding description added to Supplementary Note 2 (Lines 91–92).

Reviewer #3

[Comment 0]

The authors have adequately addressed all of my previous concerns. In particular, the newly added experiment on ultrasound-encoded audio recovery provides an important and convincing validation of the proposed method and substantially strengthens the manuscript.

I am satisfied with the authors' responses and revisions. I therefore have no further concerns and recommend that the manuscript be accepted for publication.

[Response 0]

We thank the reviewer for the positive assessment and recommendation for publication. We appreciate the recognition of our efforts to perform a new experiment on ultrasound-encoded audio recovery, which further strengthens the manuscript. We are pleased that the revision addresses all concerns.
