# Supplementary material for: Ultrahigh-speed chromatically band-limited illumination profilometry
Source: Nat Commun. 2026 Oct 12;17:11567. doi: 10.1038/s41467-026-78478-w (PMC13748529; doi:10.1038/s41467-026-78478-w)
Supplement: Supplementary file 2 [file 41467_2026_78478_MOESM1_ESM.pdf]

SUPPLEMENTARY MATERIALS

Ultrahigh-speed chromatically band-limited illumination profilometry

Cheng Jiang[1] and Jinyang Liang[1,*]

[1]Laboratory of Applied Computational Imaging, Centre Énergie Matériaux Télécommunications, Institut National de la Recherche Scientifique, Université du Québec, 1650 boulevard Lionel-Boulet, Varennes, Québec J3X1P7, CANADA

[*]Corresponding author: jinyang.liang@inrs.ca

Supplementary Note 1: Overview of fringe projection profilometry (FPP)

Fringe projection profilometry (FPP) is one of the most widely used three-dimensional (3D) surface imaging modalities. Driven by the demand for speed, accuracy, and robustness, FPP has diversified into a broad family of methods, which can be distinguished, in general, by how fringes are generated and how phase is extracted.

Early FPP systems generated fringes with a physical, fixed grating. For example, moiré profilometry, introduced by Takasaki [1], places a grating close to the object and views it through the same grating, so that the grating lines and their shadows cast on the surface form moiré contour fringes whose phase encodes depth. A fixed grating, however, locks the fringe pitch in hardware and limits pattern flexibility. To overcome the restriction, Li et al. introduced computer-generated moiré profilometry [2], forming the moiré pattern numerically through digital fringe superposition. Meanwhile, the rapid development in the projector industry enabled user-defined fringe display at video rate. Zhang et al., for instance, developed real-time profilometry by using bicolor grating video projection [3]. This programmability and flexibility now underpin most FPP methods [4,5].

Once projected onto a 3D object, fringes are deformed by its surface, and the deformed fringe images are recorded by a camera. Surface information is then recovered from the phase of these deformed fringes. Depending on the number of acquired frames, existing techniques can be generally categorized into multi-frame and single-frame approaches. Multi-frame methods extract phase from a sequence of fringe images. For example, phase-shifting profilometry projects several fringe patterns with known phase steps and solves the depth-encoded phase point-by-point, offering high accuracy and strong noise immunity [6-8]. Several variants are built on this phase-shifting scheme. For instance, Su et al. used modulation measuring profilometry [9], which scans the object along the optical axis and determines surface height where the fringe contrast reaches its

maximum. Another example is phase-differencing profilometry proposed by Wei et al., which directly solves for the phase difference between the 3D object and the reference plane [10]. Moreover, Xu et al. combined adaptive fringe projection with curve fitting to extend the dynamic range [11], and Wang et al. corrected saturation-induced phase errors using inverted fringes [12,13]. Deep learning has been implemented in multi-frame-based phase extraction [14]. For example, Zhang et al. used a U-Net to recover missing fringe information in high-reflectivity regions [15]. Wang et al. combined a generative adversarial network with a U-Net for 3D recovery of surfaces with large reflectivity differences [16]. Tan et al. applied deep Bayesian inference to translucent media, for which conventional demodulation fails due to surface and subsurface scattering that corrupts the fringes [17].

Single-frame methods extract the phase from one fringe image. For example, Zheng et al. proposed empirical mode decomposition-based profilometry [18], which adaptively decomposes a single fringe image into intrinsic mode functions, discards the low-frequency background and noise, and retrieves phase from the isolated carrier by Hilbert transform. Wang et al. combined two-dimensional wavelet transform profilometry with spatial phase unwrapping to reconstruct continuously deformed objects from a single fringe pattern [19]. Another important method in this category is Fourier transform profilometry (FTP), introduced by Takeda et al. [20]. FTP isolates the carrier in the frequency domain by band-pass filtering and retrieves phase by inverse Fourier transforming the filtered spectrum shifted to the center. Deep learning has further improved single-frame phase extraction. Feng et al. trained a deep network to predict the numerator and denominator terms of the arctangent from a single fringe pattern to compute the phase [21]. They later added uncertainty estimation, outputting a per-pixel confidence map alongside the phase to identify where the prediction is unreliable [22]. Qin et al. demonstrated reliable phase extraction by restoring high-quality fringes from a single composite pattern with a multi-attention network [23]. Ji et al. used two parallel U-Nets to predict the wrapped phase and coarse depth from a single fringe image for accurate depth reconstruction [24].

Supplementary Note 2: Phase extraction in FPP

The arctangent operation [Eq. (5) in Main Text] confines the result to $(-\pi, \pi]$, producing a wrapped phase. Extracting the continuous phase therefore requires phase unwrapping—an indispensable

step in FPP. Existing methods fall into two categories, temporal phase unwrapping (TPU) and spatial phase unwrapping (SPU), distinguished by whether the fringe order is inferred from multiple patterns acquired over time or from spatial neighbors within a single wrapped map.

TPU determines the fringe order at each pixel from a series of patterns projected over time [25]. One class of TPU resolves the order using fringes of different frequencies. For example, Li et al. proposed a multi-frequency hierarchical approach that uses a low-frequency phase to guide the unwrapping of a high-frequency phase [26]. As another example, the dual-wavelength heterodyne approach uses the beating of two phases of close frequencies to synthesize a longer equivalent wavelength whose single period spans the full field, so that the fringe order of the high-frequency phase can be determined unambiguously [27]. Recent efforts focus on decreasing the number of projection patterns. To this end, Wang et al. reduced three-frequency TPU from nine to six projected fringes by combining two-plus-one profilometry with Hilbert-transform phase retrieval [28]. Yuan et al. achieved single-frequency TPU without auxiliary patterns by using a cyclic phase-shifting sequence for fringe-order mapping [29]. A second class embeds an explicit code that marks the order directly. A classical binary-coding scheme is the Gray-code method [30], and later variants reduce the number of auxiliary patterns or improve order reliability, including space-division multiplexing binary coding [31], phase coding [32], phase-shift coding with interleaved projection [33] and its division-multiplexing form [34], fringe-amplitude encoding [35], phase-intensity hybrid encoding [36], spatial symmetric multi-level Gray coding [37], and the order-shifting scheme proposed by Qin et al. [38]. Across all these approaches, multiple patterns must be projected and captured for reconstruction, so the robustness of absolute-phase recovery comes at the expense of temporal resolution.

In contrast, SPU recovers the phase from a single wrapped map by assuming surface continuity: adjacent pixels are taken to differ by less than π, and integer multiples of 2π are accumulated along an unwrapping path. Unlike TPU, SPU does not require multi-frame acquisition over time. The methods differ mainly in how the unwrapping path is chosen so that errors do not spread. Reliability-guided methods order the path from high- to low-reliability pixels [39], while scanning-based variants impose particular path geometries, such as the diamond-type [40] and curtain-type [41] strategies. As another example, multi-anchor bidirectional localization allows suppressing path-dependent error propagation in line-scanning SPU [42]. Branch-cut methods take a different route, detecting residues and connecting them with cuts that the unwrapping path may

not cross, thereby confining errors and yielding a reliable fringe order across connected regions [43]. The well-established 2D Goldstein branch-cut algorithm [44], which determines branch-cut placement by iteratively connecting nearby phase residues with opposite signs, was selected for C-BLIP phase recovery.

Supplementary Note 3: Mechanical assembly of the projection module of the chromatically band-limited illumination profilometry (C-BLIP) system

To support the compact layout and easy alignment of the chromatically band-limited illumination profilometry (C-BLIP, Fig. 1 in Main Text) system, we designed and 3D-printed custom components of its projection module (Supplementary Fig. 2a). All CAD sources and printable files for the 3D-printed mounting base are accessible online [45]. Major assembly steps are listed below:

Step 1: As shown in Supplementary Fig. 2b, each high-power LED chip is mounted onto an aluminum heatsink to dissipate heat. These units are housed in a mount, providing wire access.

Step 2: A collimation lens is inserted into a housing for each LED (Supplementary Fig. 2c) and fixed into a dove-tailed mount.

Step 3: Shown in Supplementary Fig. 2d, an X-cube beam combiner is secured in a holder, ensuring angular and translational stability.

Step 4: Displayed in Supplementary Fig. 2e, a relay lens, which relays the combined beam onto the digital micromirror device (DMD), is mounted in the same manner as shown in Supplementary Fig. 2c.

Step 5: A mirror is mounted with a compact kinematic mirror mount (Thorlabs, KMS) and secured in a holder (Supplementary Fig. 2f).

Step 6: A modular mount integrates a total-internal-reflection (TIR) prism to a DMD with fixed alignment tolerances. The DMD is fixed using a mechanical holder (Supplementary Fig. 2g) to ensure stability.

Step 7: All components are anchored onto a base (Supplementary Fig. 2h), which is a monolithic platform that offers pre-calibrated socketing for each optical and electronic component. This approach enables repeatable assembly, reduces alignment errors, and reduces the

system footprint to 21 cm × 22 cm without compromising the optical and thermal performance.

Supplementary Note 4: Calibration of C-BLIP

The transformation from the world coordinate system to the camera/projector coordinate systems can be mathematically described by the pinhole model, which provides the solution for the triangulation method [46]. For a 3D point (*x, y, z*) in the world coordinate system, in a perfect imaging condition, the pinhole model describes its corresponding pixel on the camera sensor, $(u_\mathrm{c}, v_\mathrm{c})$, to be

$$s_\mathrm{c}\begin{bmatrix}u_\mathrm{c}\\ v_\mathrm{c}\\ 1\end{bmatrix} = A_\mathrm{c}[R_\mathrm{c}\ T_\mathrm{c}]\begin{bmatrix}x\\ y\\ z\\ 1\end{bmatrix} = \begin{bmatrix}f_{cu} & 0 & u_\mathrm{cp}\\ 0 & f_{cv} & v_\mathrm{cp}\\ 0 & 0 & 1\end{bmatrix}[R_\mathrm{c}\ T_\mathrm{c}]\begin{bmatrix}x\\ y\\ z\\ 1\end{bmatrix}. \tag{S1}$$

Akin to the pinhole camera model, the projective behavior of the projector can also be described based on the corresponding projector pixel $(u_\mathrm{p}, v_\mathrm{p})$ as

$$s_\mathrm{p}\begin{bmatrix}u_\mathrm{p}\\ v_\mathrm{p}\\ 1\end{bmatrix} = A_\mathrm{p}[R_\mathrm{p}\ T_\mathrm{p}]\begin{bmatrix}x\\ y\\ z\\ 1\end{bmatrix} = \begin{bmatrix}f_{\mathrm{p}u} & 0 & u_\mathrm{pp}\\ 0 & f_{\mathrm{p}v} & v_\mathrm{pp}\\ 0 & 0 & 1\end{bmatrix}[R_\mathrm{p}\ T_\mathrm{p}]\begin{bmatrix}x\\ y\\ z\\ 1\end{bmatrix}. \tag{S2}$$

In Eqs. (S1)–(S2), the matrices A_c and A_p contain the intrinsic parameters of the camera and projector: (f_{cu}, f_{cv}) and $(f_{\mathrm{p}u}, f_{\mathrm{p}v})$ describe the effective focal lengths along the axes of the camera and projector; $(u_\mathrm{cp}, v_\mathrm{cp})$ and $(u_\mathrm{pp}, v_\mathrm{pp})$ are the coordinates of the principal points of the camera and projector, respectively. Defined as the extrinsic parameters of the camera and projector, R_c and R_p are 3×3 matrices accounting for rotation. T_c and T_p are 3×1 matrices for translation. Here, we define $A_\mathrm{c}[R_\mathrm{c}\ T_\mathrm{c}] = \begin{bmatrix}a_{11} & \cdots & a_{14}\\ \vdots & \ddots & \vdots\\ a_{31} & \cdots & a_{34}\end{bmatrix}$ and $A_\mathrm{p}[R_\mathrm{p}\ T_\mathrm{p}] = \begin{bmatrix}b_{11} & \cdots & b_{14}\\ \vdots & \ddots & \vdots\\ b_{31} & \cdots & b_{34}\end{bmatrix}$. Finally, s_c and s_p are scalar factors for numerical extraction of $(u_\mathrm{c}, v_\mathrm{c})$ and $(u_\mathrm{p}, v_\mathrm{p})$.

The intrinsic and extrinsic parameters can be extracted by system calibration. For camera calibration, a flat checkerboard calibration plane was used. The flat checkerboard positioned with different poses was imaged by the camera. All captured checkerboard images were processed using the open-source MATLAB toolbox to extract the grid corners and calculate the camera's calibration parameters [47].

For projector calibration, the projector was modeled as a virtual camera [48]. A phase-shifted fringe set with a total phase shift of 2π was projected for each fringe orientation, together with its orthogonal counterpart. The unwrapped phase maps from the two orthogonal fringe directions provided independent measurements of the projector's two-dimensional pixel indices, enabling accurate calibration of the stereo geometry between the physical camera and the virtual projector.

Supplementary Note 5: System synchronization and data acquisition of C-BLIP

Supplementary Figs. 3c and d illustrate the precise timing relationships among DMD pattern display, LED illumination, and camera exposures that enable C-BLIP's continuous high-speed operation for both the intensity 3D imaging mode and the color 3D imaging mode. Accordingly, the specifications of the DMD, camera resolution, time scale of system operation, and field of view (FOV) are summarized in Supplementary Table 2 and explained below.

The intensity 3D imaging mode triggers LEDs to illuminate sequentially (Supplementary Fig. 3c). In the 75-kfps intensity 3D imaging mode, the DMD cycles through four binary fringe patterns of distinct orientations (i.e., Fringes 1 to 4) at 25 kHz. Each 40-μs pattern interval consists of t_{D} = 35 μs for pattern display and 5 μs for DMD resetting. The RGB LEDs are temporally multiplexed within each pattern interval: each LED is turned on for $t_{\mathrm{L}} = 11.6$ μs (one-third of the t_{D} = 35 μs display time) while the other two remain off. The camera's exposure time t_{e} = 160 μs is aligned to capture one full DMD cycle (i.e., four patterns). The DMD's active region remains 160×1140 pixels, giving the same field of view of 13.5×48.1 cm².

In the 150-kfps intensity 3D imaging mode, the system operates similarly to the 75-kfps mode but with a higher speed. The DMD refreshes at 50 kHz, and each LED is sequentially illuminated for $t_{\mathrm{L}} = 5$ μs (one-third of the t_{D} = 15 μs display time). The camera exposure time is reduced to $t_{\mathrm{e}} = 80$ μs to match the faster cycling. In this case, the DMD's active region is reduced to 80×1140 pixels, resulting in a smaller field of view of 3.5×24.9 cm².

In the color 3D imaging mode (Supplementary Fig. 3d), the DMD operates at 25 kHz with the same fringe sequence. Accordingly, in each DMD pattern display period, the red, green, and blue LEDs illuminate for a time of $t_{\mathrm{L}} = t_{\mathrm{D}} = 35$ μs. The camera is continuously exposed with $t_{\mathrm{e}} = 160$ μs. In this mode, the DMD's active region is 160×1140 pixels, corresponding to a field of view of 13.5×48.1 cm².

Supplementary Note 6: Depth resolution quantification of C-BLIP

To quantify C-BLIP's depth resolution, we imaged a flat reference plate (35 mm × 35 mm in size) under the operating modes by following a commonly used plane-fitting evaluation procedure [49,50]. Displayed in Supplementary Fig. 4, in each case, we reconstructed the surface via C-BLIP, then performed a least-squares fit of the point cloud to an ideal plane. The per-point deviation from that fitted plane yielded an error map, and its root mean square error (RMSE) is calculated. For each operating mode, the reported depth resolution is the mean of the RMSE values obtained from the reconstructions corresponding to all demultiplexed fringe images (four in color-3D-imaging mode and 12 in intensity-3D-imaging mode), and the associated standard deviation represents the variation among these RMSE values. The mean absolute error (MAE) is also reported. For the 25-kfps color 3D imaging operation, the depth resolution was quantified as 0.70 ± 0.08 mm (mean ± standard deviation), with an MAE of 0.59 ± 0.06 mm. For 75-kfps intensity 3D imaging, the depth resolution was 0.93 ± 0.14 mm, with an MAE of 0.77 ± 0.10 mm, and for 150-kfps intensity 3D imaging, the depth resolution was 1.10 ± 0.18 mm, with an MAE of 0.90 ± 0.14 mm.

Supplementary Note 7: Details of message-to-sound encryption in C-BLIP

For message-encrypted spectrogram synthesis, we begin by encoding the entire signal as a 2D matrix of spectral amplitude levels $A_{m,n} = A(F_m, t_n)$ with the size of 129 × 1275 elements. Here, $m \in \{0, 1, \dots, 128\}$ rows arise from our choice of a 256-sample FFT on signals sampled at F_s =150 kHz. A one-sided transform yields 129 unique frequency bins, each spaced by 0.59 kHz over the 0–75 kHz range. The $n \in \{0, 1, \dots, 1274\}$ columns correspond to time units. They divide the 8.5-ms recording time, which corresponds to the full duration of the encrypted Morse-code message, into 1275 time slots with each time unit of $\Delta t = \frac{1}{F_\mathrm{s}}$.

The amplitude level of the audible-cover-tone frequency 10 kHz was set to 0 dB for the entire recording time. When encoding a dot or dash, we additionally set the amplitude levels for 25 kHz or 30 kHz to 0 dB. The amplitude level of all other bins stayed at -30 dB. Such a mechanism generated the spectral amplitude level maps as shown in Supplementary Fig. 5a. Once this matrix was fully populated with preset dB values, we converted it to a continuous time-domain waveform

at a sampling rate of 150 kHz using additive sinusoidal synthesis [51]. The time-domain signal is calculated as

$$R_n = \sum_{m:\, A_{m,n=0\ \mathrm{dB}}} 10^{\frac{A_{m,n}}{20}} \sin(2\pi F_m t_n), \tag{S3}$$

where $t_n = n\Delta t$. Following this manner, the encoded spectral amplitude level maps of "LACI" and its complementary counterpart are converted to time-domain signals, as shown in Supplementary Fig. 5b.

Supplementary Note 8: Details of ultrasound-encoded audio recovery using C-BLIP

The spoken word "Hi" was recorded by a cell phone as a 250-ms waveform. The recording was dc-removed and amplitude-normalized, which was used as the ground truth (Fig. 6b in Main Text). This baseband audio, denoted by $s_\mathrm{A}(t)$, was then encoded onto an ultrasonic carrier by amplitude modulation, with a carrier frequency of f_C = 25 kHz. The modulation follows [52]

$$s_\mathrm{AM}(t) = [1 + m s_\mathrm{A}(t)]\sin(2\pi f_\mathrm{C} t), \tag{S4}$$

with a modulation index $m = 1.0$ [Ref. 53]. Since the $s_\mathrm{A}(t)$ spans from 0 to 4 kHz (i.e., bandwidth B_A=4 kHz), the modulation copies the speech onto the upper and lower sidebands at $f_\mathrm{C} \pm B_\mathrm{A}$, placing its information in the 21–29 kHz band.

The reconstructed depth from C-BLIP, averaged over the central region of the membrane, gives a time-resolved trace $z(t)$ that carries the acoustic-induced vibration (Fig. 6e in Main Text). Then, the audio was recovered from this trace alone by single-sideband demodulation [54]. The analytic signal was therefore formed by

$$z_\mathrm{analytic}(t) = z(t) + i\mathcal{H}\{z(t)\}. \tag{S5}$$

where $\mathcal{H}\{\cdot\}$ denotes the Hilbert transform. From $z_\mathrm{analytic}(t)$, the upper sideband $z_\mathrm{usb}(t)$ was then isolated by using a fourth-order Butterworth band-pass filter [55]. The lower bound was set 0.1 kHz above the carrier to exclude the strong carrier line at f_C, and the upper bound at 4 kHz matches the speech bandwidth. The isolated sideband was then shifted down to the baseband by

$$b(t) = z_\mathrm{usb}(t) e^{-i2\pi f_\mathrm{C} t}. \tag{S6}$$

The audio was recovered by taking the real part of the complex baseband signal as

$$\hat{s}_\mathrm{A}(t) = \Re[b(t)]. \tag{S7}$$

Finally, the recovered waveform was compared with the ground-truth recording in both the time and frequency domains, evaluated over the whole interval. The agreement between the recovered and the ground-truth traces was quantified by the normalized RMSE [56] that divides the standard RMSE value by the signal range, with the number of temporal samples of $0.25\ \text{s} \times 150\ \text{kfps} = 37500$.

Supplementary References

1 Takasaki, H. Moiré topography. *Applied Optics* **9**, 1467-1472 (1970).

2 Li, C. *et al.* Computer-generated moiré profilometry based on fringe-superposition. *Scientific Reports* **10**, 17202 (2020).

3 Zhang, Y., Cao, Y., Wu, H., An, H. & Huang, X. Real-time profilometry by bicolor grating video projection. *Plos One* **16**, e0259569 (2021).

4 Feng, S. *et al.* High dynamic range 3D measurements with fringe projection profilometry: a review. *Measurement Science and Technology* **29**, 122001 (2018).

5 Zhang, S. Recent progresses on real-time 3D shape measurement using digital fringe projection techniques. *Optics and Lasers in Engineering* **48**, 149-158 (2010).

6 Zuo, C. *et al.* Phase shifting algorithms for fringe projection profilometry: a review. *Optics and Lasers in Engineering* **109**, 23-59 (2018).

7 Huang, P., Zhang, S. & Chiang, F.-P. Trapezoidal phase-shifting method for three-dimensional shape measurement. *Optical Engineering* **44**, 123601 (2005).

8 Jia, P., Kofman, J. & English, C. Multiple-step triangular-pattern phase shifting and the influence of number of steps and pitch on measurement accuracy. *Applied Optics* **46**, 3253-3262 (2007).

9 Su, L., Su, X., Li, W. & Xiang, L. Application of modulation measurement profilometry to objects with surface holes. *Applied Optics* **38**, 1153-1158 (1999).

10 Wei, Z. *et al.* Dynamic phase-differencing profilometry with number-theoretical phase unwrapping and interleaved projection. *Optics Express* **32**, 19578-19593 (2024).

11 Xu, P., Liu, J. & Wang, J. High dynamic range 3D measurement technique based on adaptive fringe projection and curve fitting. *Applied Optics* **62**, 3265-3274 (2023).

12 Wang, J., Yang, Y., Xu, P. & Liu, J. Saturation-induced phase error correction method in 3-D measurement based on inverted fringes. *Applied Optics* **62**, 492-499 (2023).

13 Wang, J. & Yang, Y. A new method for high dynamic range 3D measurement combining adaptive fringe projection and original-inverse fringe projection. *Optics and Lasers in Engineering* **163**, 107490 (2023).

14 Zuo, C. *et al.* Deep learning in optical metrology: a review. *Light: Science & Applications* **11**, 39 (2022).

15 Zhang, L., Ji, Y., Wu, W. & Wang, J. Deep learning-based restoration method for missing fringe information in the high reflectivity regions. *Precision Engineering* **96**, 80-93 (2025).

16 Wang, J., Zhang, S. & Yang, Y. Deep learning-based high dynamic range 3D measurement: the combination of GAN and U-Net. *Sensors and Actuators A: Physical* **399**, 117405 (2026).

17 Tan, J., Niu, H., Su, W. & He, Z. Structured light 3D shape measurement for translucent media base on deep Bayesian inference. *Optics & Laser Technology* **181**, 111758 (2025).

18 Zheng, S. & Cao, Y. Fringe-projection profilometry based on two-dimensional empirical mode decomposition. *Applied Optics* **52**, 7648-7653 (2013).

19 Wang, J., Yang, Y. & Zhou, Y. Dynamic three-dimensional surface reconstruction approach for continuously deformed objects. *IEEE Photonics Journal* **13**, 1-15 (2021).

20 Takeda, M. & Mutoh, K. Fourier transform profilometry for the automatic measurement of 3-D object shapes. *Applied Optics* **22**, 3977-3982 (1983).

21 Feng, S. *et al.* Fringe pattern analysis using deep learning. *Advanced Photonics* **1**, 025001 (2019).

22 Feng, S., Zuo, C., Hu, Y., Li, Y. & Chen, Q. Deep-learning-based fringe-pattern analysis with uncertainty estimation. *Optica* **8**, 1507-1510 (2021).

23 Qin, J., Jiang, Y., Cao, Y. & Wu, H. Single-shot phase-shifting composition fringe projection profilometry by multi-attention fringe restoration network. *Neurocomputing* **634**, 129908 (2025).

24 Ji, Y., Zhang, L., Wu, W. & Wang, J. Deep learning-based single-frame depth reconstruction empowered by indirect mapping. *IEEE Transactions on Instrumentation and Measurement* **74**, 7010711 (2025).

25 Zuo, C., Huang, L., Zhang, M., Chen, Q. & Asundi, A. Temporal phase unwrapping algorithms for fringe projection profilometry: a comparative review. *Optics and Lasers in Engineering* **85**, 84-103 (2016).

26 Li, E. *et al.* Multi-frequency and multiple phase-shift sinusoidal fringe projection for 3D profilometry. *Optics Express* **13**, 1561-1569 (2005).

27 Song, L. *et al.* Phase unwrapping method combining dual-frequency heterodyne with dual complementary phase encoding. *Optical Engineering* **60**, 085106 (2021).

28 Wang, J., Ji, Y., Zhang, L. & Wu, W. Fringe projection 3D imaging optimized for accuracy and efficiency. *Optics Express* **33**, 54709-54732 (2025).

29 Yuan, H. *et al.* Single-frequency temporal phase unwrapping without additional patterns for 3D shape measurement. *Optics & Laser Technology* **204**, 115951 (2026).
30 He, X. & Kemao, Q. A comparison of n-ary simple code and n-ary gray code phase unwrapping in high-speed fringe projection profilometry. *Optics and Lasers in Engineering* **128**, 106046 (2020).
31 Wu, H., Cao, Y., Dai, Y. & Zhang, H. Ultra-fast 3D imaging by a big codewords space division multiplexing binary coding. *Optics Letters* **48**, 2793-2796 (2023).
32 Wang, Y. & Zhang, S. Novel phase-coding method for absolute phase retrieval. *Optics Letters* **37**, 2067-2069 (2012).
33 An, H., Cao, Y., Li, H. & Zhang, H. High-speed 3-D reconstruction based on phase shift coding and interleaved projection. *Expert Systems with Applications* **234**, 121067 (2023).
34 Wu, F., Cao, Y., An, H. & Wei, Z. Dynamic three-dimensional reconstruction with phase shift coding division multiplexing. *Sensors and Actuators A: Physical* **379**, 115847 (2024).
35 Wang, J., Cao, Y., Wu, H. & Wei, Z. Absolute phase retrieval based on fringe amplitude encoding without any additional auxiliary pattern. *Optics Express* **31**, 41952-41966 (2023).
36 Luo, J., Cao, Y. & Wang, J. Absolute phase retrieval based on phase-intensity hybrid encoding without any additional pattern. *Optics and Lasers in Engineering* **190**, 108968 (2025).
37 Li, Y., Cao, Y. & Ran, L. Spatial symmetric multi-level coding absolute phase retrieval for color 3D real-time measurement. *Measurement* **257**, 119017 (2025).
38 Qin, J., Jiang, Y. & Cao, Y. Order-shifting temporal phase unwrapping using uneven duty gray-code for high-accurate and high-efficient 3D measurement. *Optics Letters* **51**, 1472-1475 (2026).
39 Su, X. & Chen, W. Reliability-guided phase unwrapping algorithm: a review. *Optics and Lasers in Engineering* **42**, 245-261 (2004).
40 Jiang, H., Xu, Y., Zhang, C., Xu, Z., Huang, J., Tan, H. & Lu, J. An algorithm combining the branch-cut method and rhombus phase unwrapping algorithm. Journal of Physics: Conference Series 1634, 012068 (2020).
41 Xu, C. *et al.* Curtain-type phase unwrapping algorithm. *Optical Engineering* **61**, 044103 (2022).
42 Zhang, Y., Cao, Y., Wu, H. & Li, C. Spatial phase unwrapping algorithm based on multi-anchors bidirectional location and suppression. *Optical Engineering* **63**, 014101 (2024).
43 De Souza, J., Oliveira, M. & Dos Santos, P. Branch-cut algorithm for optical phase unwrapping. *Optics Letters* **40**, 3456-3459 (2015).
44 Goldstein, R. M., Zebker, H. A. & Werner, C. L. Satellite radar interferometry: two-dimensional phase unwrapping. *Radio Science* **23**, 713-720 (1988).
45 Jiang, C. & Liang, J. Ultrahigh-speed chromatically band-limited illumination profilometry. Zenodo (2025). https://doi.org/10.5281/zenodo.17468916
46 Jiang, C., Kilcullen, P., Lai, Y., Ozaki, T. & Liang, J. High-speed dual-view band-limited illumination profilometry using temporally interlaced acquisition. *Photonics Research* **8**, 1808-1817 (2020).
47 Bouguet, J.-Y. Camera calibration toolbox for matlab. *http://www.vision.caltech.edu/bouguetj/calib_doc/* (2004).
48 Zhang, S. & Huang, P. S. Novel method for structured light system calibration. *Optical Engineering* **45**, 083601-083608 (2006).
49 Rao, G. *et al.* Depth-driven variable-frequency sinusoidal fringe pattern for accuracy improvement in fringe projection profilometry. *Optics Express* **26**, 19986-20008 (2018).
50 Song, H., Kong, L., Tang, X., Xu, X. & Wang, B. Extrinsic parameters optimization for fringe projection system based on standard components. *Measurement* **242**, 115836 (2025).
51 Serra, X. & Smith, J. Spectral modeling synthesis: a sound analysis/synthesis system based on a deterministic plus stochastic decomposition. *Computer Music Journal* **14**, 12-24 (1990).
52 Haykin, S. *Communication systems*. (John Wiley & Sons, 2008).
53 Lathi, B. P. & Ding, Z. *Modern digital and analog communication systems*. Vol. 3 (Oxford university press New York, 1998).
54 Weaver, D. K. A third method of generation and detection of single-sideband signals. *Proceedings of the IRE* **44**, 1703-1705 (1956).
55 Butterworth, S. On the theory of filter amplifiers. *Wireless Engineer* **7**, 536-541 (1930).
56 Kang, E., Min, J. & Ye, J. C. A deep convolutional neural network using directional wavelets for low-dose X-ray CT reconstruction. *Medical Physics* **44**, e360-e375 (2017).

Supplementary Figures

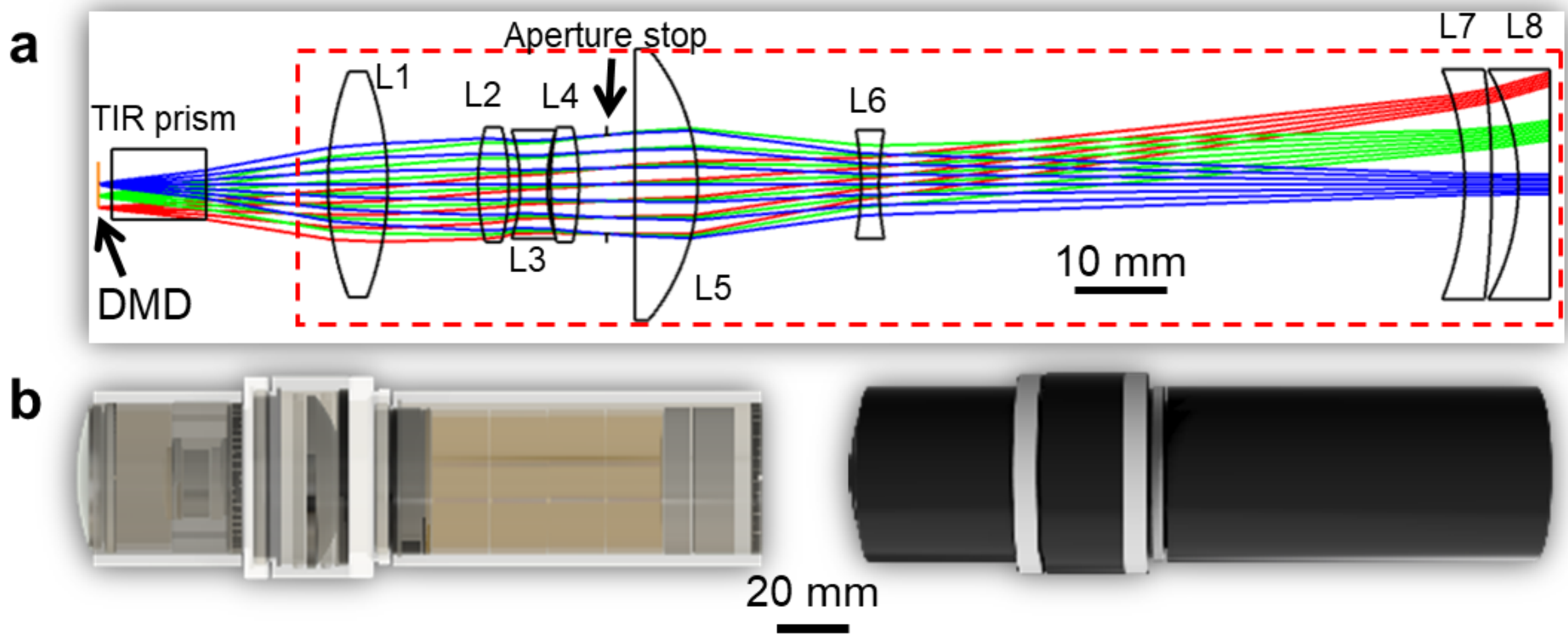

Supplementary Fig. 1. Design of the chromatically band-limited projection lens. a Optical layout and ray trace from the digital micromirror device (DMD) through the projection lens (marked by the red dashed box). Ray colors indicate different field positions. TIR: total internal reflection. This figure was generated using Ansys Zemax OpticStudio and is used courtesy of ANSYS, Inc. **b** Transparent (left) and opaque (right) views of the designed projection lens. The CAD models are courtesy of Thorlabs, Inc. (for the lenses, lens tubes, optic spacers, and mount adapters) and Edmund Optics, Inc. (for the lenses).

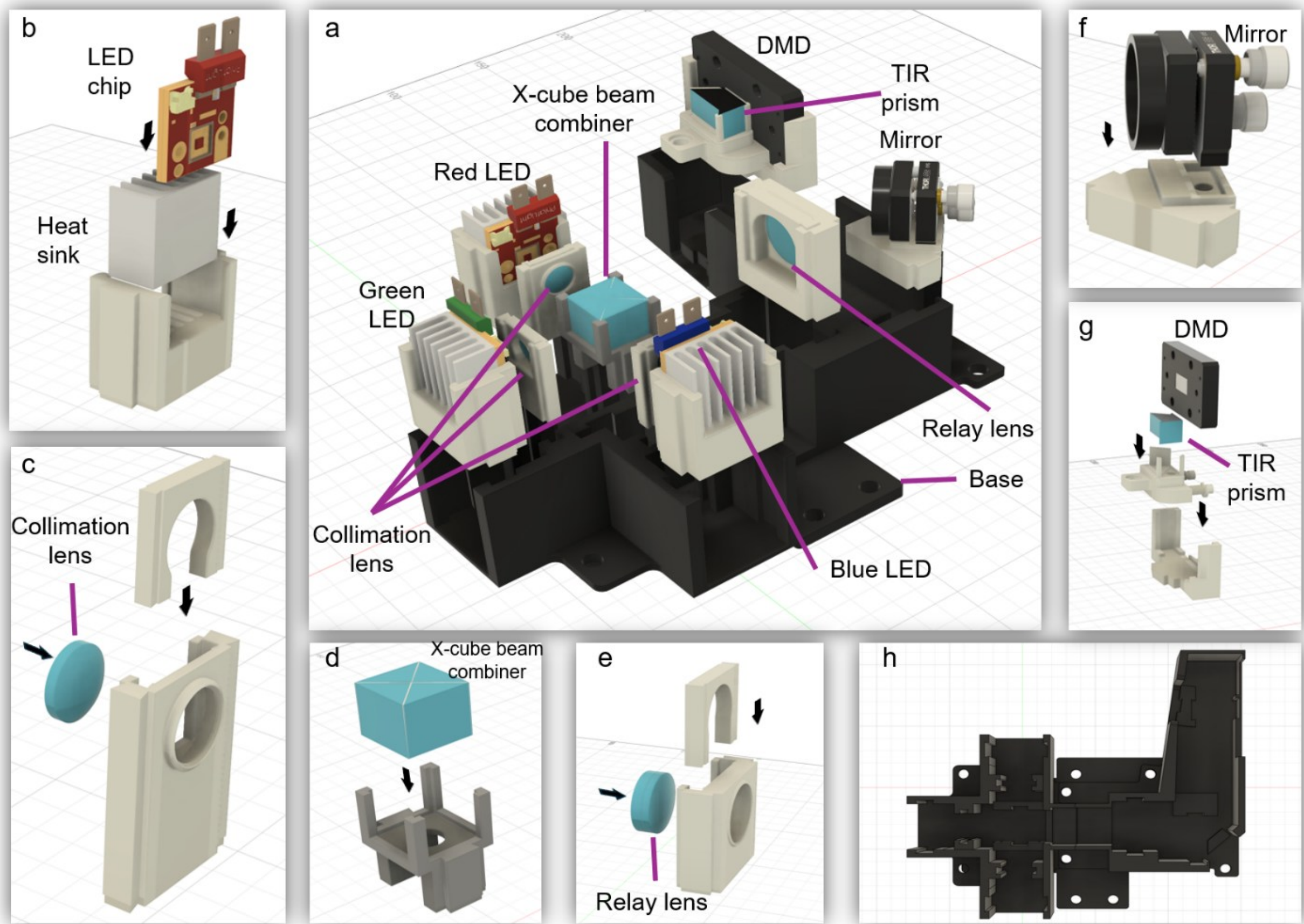

Supplementary Fig. 2. Mechanical assembly of the projection module of the chromatically band-limited illumination profilometry (C-BLIP) system. a Overview of the designed C-BLIP projection module. **b** Assembly of the chip of the red, green, or blue light-emitting diode (LED) and its heatsink mount. **c** Assembly of the collimation lens and its housing. **d** Assembly of the X-cube beam combiner and its holder. **e** Assembly of the relay lens and its housing. **f** Assembly of the mirror, the mirror mount, and the base. **g** Assembly of the TIR prism and the DMD mounting base. **h** Top view of the base supporting all modules. Arrows indicate the assembly directions. The CAD models are courtesy of Luminus Devices, Inc. (for the LEDs) and Thorlabs, Inc. (for the mirror mount, collimation lens, and relay lens).

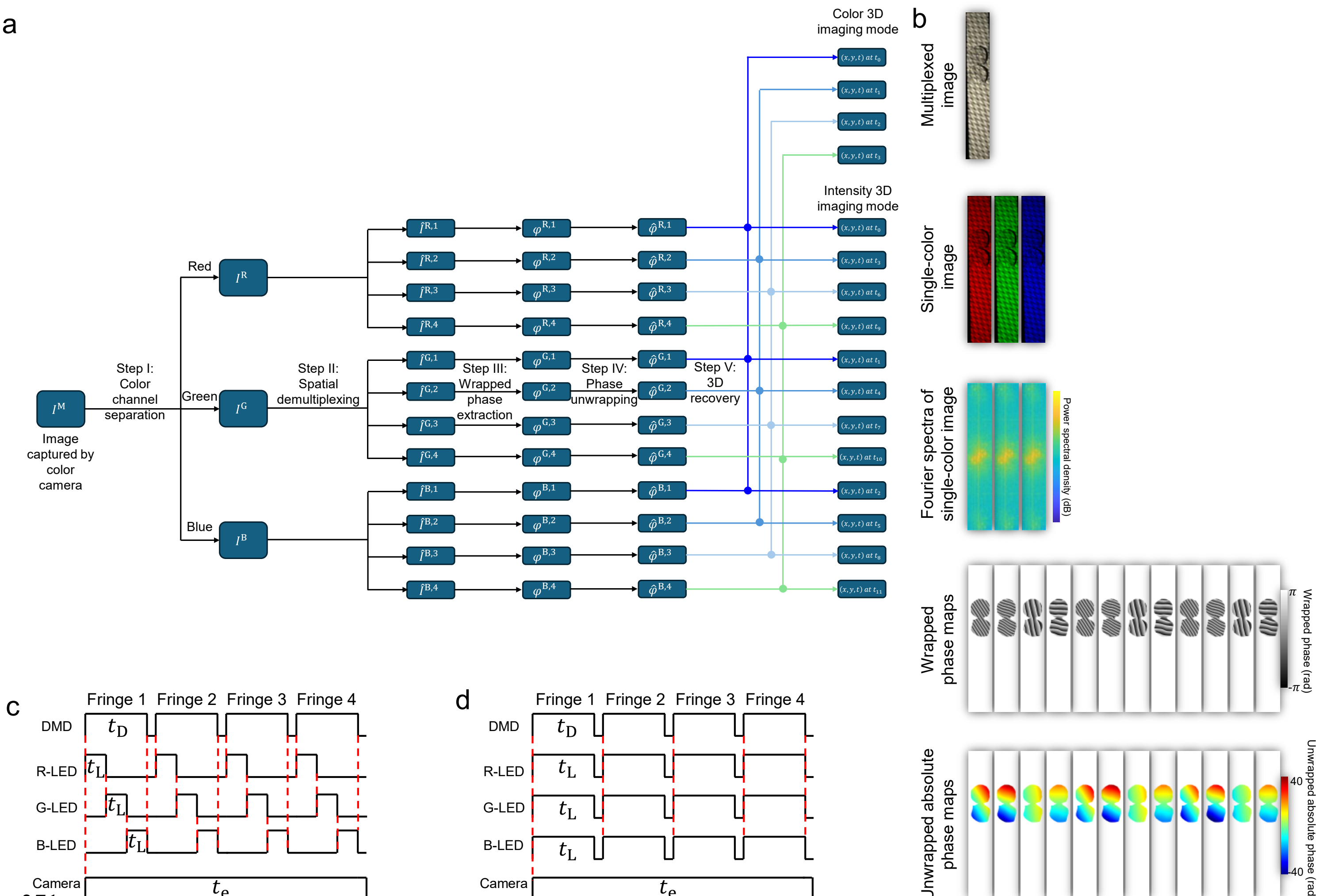

Supplementary Fig. 3. Image reconstruction of C-BLIP. a Flowchart. I^{M}: multiplexed snapshot recorded by the color camera. I^{s} ($s = [\mathrm{R}, \mathrm{G}, \mathrm{B}]$): single-color images from I^{M}. $\hat{I}^{s,i}$, $\varphi^{s,i}$, and $\hat{\varphi}^{s,i}$ (i=1–4): demultiplexed fringe images, wrapped phase maps, and unwrapped absolute phase maps, respectively. (x, y, z): recovered 3D coordinates. **b** Example of image analysis for phase extraction. **c–d** Timing diagrams illustrating the synchronization of the intensity 3D imaging mode (c) and the color 3D imaging mode (d). t_{D}, t_{L}, and t_{e}: display time of the DMD, illumination time of LEDs, and exposure time of the color camera.

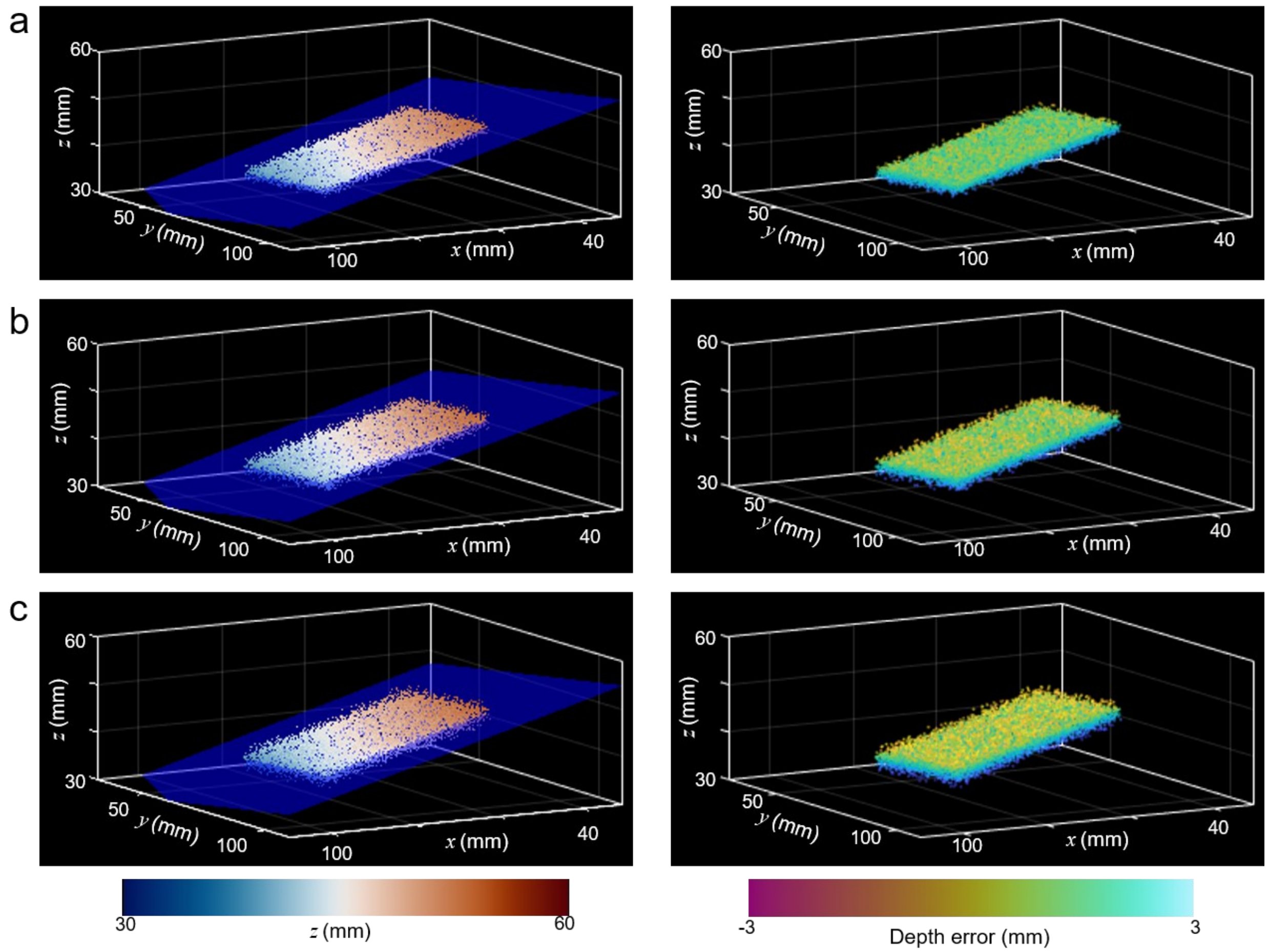

Supplementary Fig. 4. Reconstructed 3D images (left column) and their error maps (right column) of 25-kfps color 3D imaging (a), 75-kfps intensity 3D imaging (b), and 150-kfps intensity 3D imaging (c). Blue planes in the left column: fitted ideal planes.

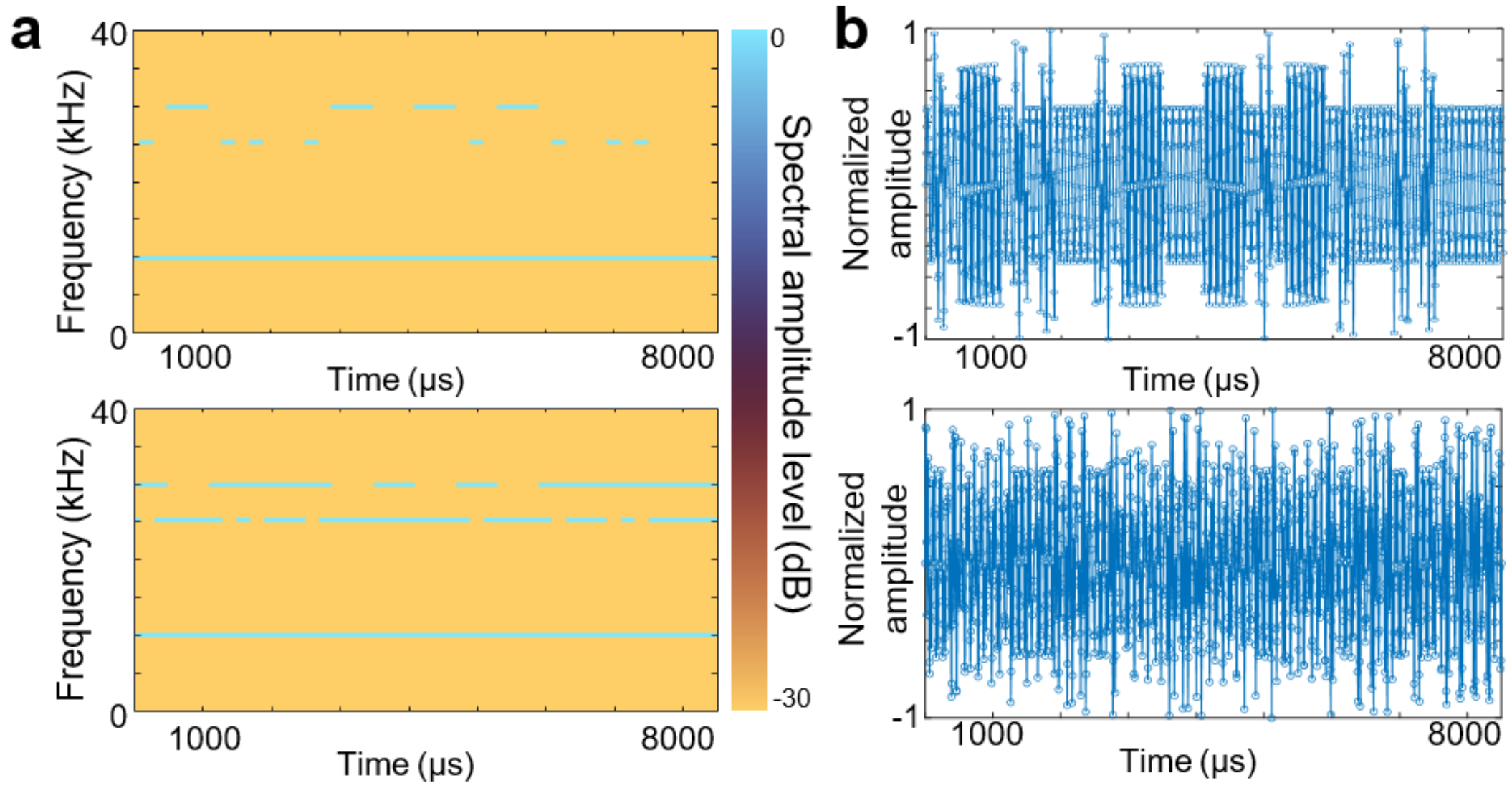

Supplementary Fig. 5. Ultrasound message encryption of C-BLIP. a Encrypted spectral amplitude level maps from "LACI" (top) and its complementary counterpart (bottom). **b** Corresponding time-domain signals generated from the encrypted spectral amplitude level maps.

Supplementary Tables

Supplementary Table 1 | Component information on the chromatically band-limited projection lens

Component / Information	L1	L2	L3	L4	Aperture stop	L5	L6	L7	L8
Vendor	Edmund Optics	Thorlabs	Edmund Optics	Thorlabs	3D printed	Edmund Optics	Edmund Optics	Thorlabs	Thorlabs
Part number	45-161	LB1014	32-988	LB1258	Dia. = 10.92 mm	48-653	48-056	LF1822	LC1715
Distance (mm)	10	1.24	0.36	3.1	3.1	18.1	64.8	3	/

Supplementary Table 2 | Specifications of C-BLIP's data acquisition

Mode	DMD pattern size (pixels)	t_{D} (µs)	t_{L} (µs)	Camera frame size (pixels)	t_{e} (µs)	Field of view (cm×cm)	Depth resolution (mm)
25-kfps color 3D imaging	160×1140	35	35	368×1472	160	13.5×48.1	0.70 ± 0.08
75-kfps intensity 3D imaging	160×1140	35	11.6	368×1472	160	13.5×48.1	0.93 ± 0.14
150-kfps intensity 3D imaging	80×1140	15	5	192×1408	80	3.5×24.9	1.10 ± 0.18
