# Supplementary material for: Ultrahigh-speed chromatically band-limited illumination profilometry
Source: Nat Commun. 2026 Oct 12;17:11567. doi: 10.1038/s41467-026-78478-w (PMC13748529; doi:10.1038/s41467-026-78478-w)
Supplement: Supplementary file 3 [file 41467_2026_78478_MOESM2_ESM.pdf]

Description of Additional Supplementary Files

Supplementary Movie 1: Animated illustration of C-BLIP's mechanical assembly and data acquisition. The CAD models are courtesy of Luminus Devices, Inc. (for the LEDs), Thorlabs, Inc. (for the mirror mount, lenses, lens tubes, optic spacers, and mount adapters), and Edmund Optics, Inc. (for the lenses).

Supplementary Movie 2: Reconstructions of optical-illusion objects with continuous viewpoint rotation, corresponding to data presented in Fig. 2.

Supplementary Movie 3: Reconstructed video of a turbofan blade-projectile in the color 3D imaging mode at 25 kfps, corresponding to data presented in Fig. 3. Az and El: azimuth and elevation.

Supplementary Movie 4: Reconstructed video of falling maple samaras in the intensity 3D imaging mode at 75 kfps, corresponding to data presented in Fig. 4c. One out of every 50 measurements is shown for video-length-control purposes.

Supplementary Movie 5: Reconstructed video of falling maple samara S_1 during the 250–350 ms time interval at 75 kfps. All measurements are displayed.

Supplementary Movie 6: Reconstructed video of two speaker membranes in the intensity 3D imaging mode at 150 kfps, corresponding to data presented in Fig. 5b. M1 is driven by a dual-tone beat signal, and M2 by a linear chirp. Az and El: azimuth and elevation.

Supplementary Movie 7: Reconstructed video of two speaker membranes driven by ultrasound-encrypted Morse code in the intensity 3D imaging mode at 150 kfps, corresponding to data presented in Fig. 5f.

Supplementary Movie 8: Reconstructed video of a thin suspended membrane vibrating in response to an ultrasound signal encoded with a spoken word "Hi", imaged in the intensity 3D imaging mode at 150 kfps, corresponding to data presented in Fig. 6. The ground-truth audio is played at the beginning of the movie, and the recovered audio is played at the end.

Supplementary Software Package: This supplementary software package contains the MATLAB scripts for C-BLIP's 3D image reconstruction and result visualization, as well as representative source data. The file Run-CBLIP.m uses representative data for image reconstruction and result visualization. All the other MATLAB files are the necessary calibration matrices and helper functions.
